# Spatial distributions of CD8 and Ki67 cells in the tumor microenvironment independently predict breast cancer-specific survival in patients with ER+HER2– and triple-negative breast carcinoma

**DOI:** 10.1371/journal.pone.0314364

**Published:** 2024-11-22

**Authors:** Dovile Zilenaite-Petrulaitiene, Allan Rasmusson, Ruta Barbora Valkiuniene, Aida Laurinaviciene, Linas Petkevicius, Arvydas Laurinavicius

**Affiliations:** 1 Department of Pathology and Forensic Medicine, Institute of Biomedical Sciences, Faculty of Medicine, Vilnius University, Vilnius, Lithuania; 2 National Centre of Pathology, affiliate of Vilnius University Hospital Santaros Klinikos, Vilnius, Lithuania; 3 Institute of Informatics, Faculty of Mathematics and Informatics, Vilnius University, Vilnius, Lithuania; Turun Yliopisto, FINLAND

## Abstract

**Introduction:**

Breast cancer (BC) presents diverse malignancies with varying biological and clinical behaviors, driven by an interplay between cancer cells and tumor microenvironment. Deciphering these interactions is crucial for personalized diagnostics and treatment. This study explores the prognostic impact of tumor proliferation and immune response patterns, assessed by computational pathology indicators, on breast cancer-specific survival (BCSS) models in estrogen receptor-positive HER2-negative (ER+HER2–) and triple-negative BC (TNBC) patients.

**Materials and methods:**

Whole-slide images of tumor surgical excision samples from 252 ER+HER2– patients and 63 TNBC patients stained for estrogen and progesterone receptors, Ki67, HER2, and CD8 were analyzed. Digital image analysis (DIA) was performed for tumor tissue segmentation and quantification of immunohistochemistry (IHC) markers; the DIA outputs were subsampled by hexagonal grids to assess the spatial distributions of Ki67-positive tumor cells and CD8-positive (CD8+) cell infiltrates, expressed as Ki67-entropy and CD8-immunogradient indicators, respectively. Prognostic models for BCSS were generated using multivariable Cox regression analysis, integrating clinicopathological and computational IHC indicators.

**Results:**

In the ER+HER2– BC, multivariable Cox regression revealed that high CD8+ density within the tumor interface zone (IZ) (HR: 0.26, *p* = 0.0056), low immunodrop indicator of CD8+ density (HR: 2.93, *p* = 0.0051), and low Ki67-entropy (HR: 5.95, *p* = 0.0.0061) were independent predictors of better BCSS, while lymph node involvement predicted worse BCSS (HR: 3.30, *p* = 0.0013). In TNBC, increased CD8+ density in the IZ stroma (HR: 0.19, *p* = 0.0119) and Ki67-entropy (HR: 3.31, *p* = 0.0250) were independent predictors of worse BCSS. Combining these independent indicators enhanced prognostic stratification in both BC subtypes.

**Conclusions:**

Computational biomarkers, representing spatial properties of the tumor proliferation and immune cell infiltrates, provided independent prognostic information beyond conventional IHC markers in BC. Integrating Ki67-entropy and CD8-immunogradient indicators into prognostic models can improve patient stratification with regard to BCSS.

## Introduction

Breast cancer (BC) is the most frequently diagnosed cancer and the leading cause of mortality among women globally [1]. The identification of diverse molecular subtypes of BC has revealed distinct clinical outcomes and survival probabilities among patients [2]. Clinical management of BC relies on well-established prognostic and predictive parameters such as tumor grade, tumor size, lymph node involvement, expression of estrogen and progesterone receptors (ER, PR) as well as human epidermal growth factor (HER2) status [3]. Several gene expression-based assays have been implemented to guide decisions toward more effective and personalized treatment strategies [4–6]. However, despite these advancements, approximately 20% of BC patients experience disease recurrence, with tumor progression in many cases [7]. This underscores the need to further enhance prognostic stratification to improve clinical decision-making and outcomes in BC patients.

For over four decades, the Ki67 immunohistochemistry (IHC) has played a significant role in assessing cellular proliferation in BC and other solid tumors, providing important prognostic and potentially predictive insights for antineoplastic therapies [8]. Findings by the monarchE committee [9] have demonstrated benefits of adding a cyclin-dependent kinase 4/6 inhibitor (abemaciclib) to hormone therapy in early-stage ER-positive (ER+) HER2-negative (HER2–) BC patients with lymph node involvement. The studies showed that a Ki67 proliferation index of ≥ 20% in patients receiving only endocrine therapy significantly increased the risk of recurrence within three years. As a result, in 2022, the American Society of Clinical Oncology [10] recommended adding abemaciclib in combination with endocrine therapy for such BC patients. Despite this, in 2023, the US Food and Drug Administration [11] revised its guidelines, eliminating the requirement for Ki67 testing before abemaciclib therapy. Nevertheless, the International Ki67 in BC Working Group [12] maintains its recommendation to assess Ki67 to guide adjuvant chemotherapy decisions. To address the well-known issues of variability and subjectivity in the semiquantitative assessment of Ki67, a dual threshold strategy has been proposed – ≤5% to indicate low proliferation and >30% to signify high proliferation, leaving patients with intermediate Ki67 levels without clear guidance on prognosis and therapy adjustments. Besides interobserver variability and somewhat arbitrary cutoffs for Ki67 positivity, the intratumoral heterogeneity (ITH) of Ki67 expression presents another challenge for accurate assessment and quantification [13, 14]. Studies have shown diverse distributions of Ki67 across distinct tumor regions, necessitating that assessment must be based on higher counts of cells, typically ranging from 500 to 2,000 [15]. It was additionally recommended to calculate the Ki67% within regions with the highest density of Ki67-positive cells, known as “hotspots”, rather than in randomly selected tumor areas [15]. However, the lack of an explicit and agreed definition of a hotspot in international guidelines left a gap in the methods to reproducibly assess spatial aspects of ITH.

Many studies have demonstrated that digital image analysis (DIA) methods offer a more objective and reproducible assessment of Ki67% compared to manual evaluations [16–19]. In a study of 237 BC patients without adjuvant systemic treatment, Gudlaugsson et al. [16] reported that Ki67 expression measured by DIA, rather than manual counting, provided better reproducibility and prognostic accuracy, thereby supporting the transition to DIA-based methodologies in clinical settings. Furthermore, digital technologies enable high-capacity analyses, thus opening new opportunities for the assessment of spatial aspects of biomarker expression, including the ITH of proliferative activity [19–23]. Plancoulaine et al. [20] proposed a method based on systematic hexagonal grid subsampling of DIA data to quantify the ITH by Haralick’s texture and bimodality indicators. Subsequent studies [19, 21] have highlighted the prognostic value of Ki67-ITH. Notably, in ER+HER2– BC, the texture entropy of Ki67-positive cells has been identified as an independent predictor of worse BC-specific survival (BCSS) [19]. Similarly, Lu et al. [23], in a comprehensive study of 2,081 patients with early‐stage ER+HER2− BC, reported that the Ki67 colocalization score, measured by the Shannon diversity index, could significantly stratify patients with regard to BCSS and distant metastasis-free survival, emphasizing the potential of ITH assessment over the standard Ki67 index.

The antitumor immune response within the tumor microenvironment is another increasingly recognized factor of disease behavior and response to therapy [24, 25]. Studies have demonstrated that tumor-infiltrating lymphocytes (TILs) – lymphocytes and plasma cells that have penetrated tumor tissue – serve as key biomarkers in several types of solid tumors, including BC [25, 26]. Particularly, in patients with HER2-positive (HER2+) and triple-negative BC (TNBC), a high presence of TILs, as determined by visual assessment, has been associated with improved overall and disease-free survival probabilities [27–30]. Moreover, in the context of HER2+ and TNBC, higher quantities of TILs correlate with a higher rate of pathological complete response after neoadjuvant therapy [30–32]. However, the prognostic role of TILs in luminal ER+HER2– BC remains unclear: while some studies have reported a correlation between the presence of TILs and negative prognostic factors [33–35], others have not found TILs to be prognostic in this BC subtype [36, 37]. Consequently, TILs are not used as a prognostic marker for luminal BC [37, 38].

In the current clinical practice, pathologists assess the levels of TIL infiltration by visually examining hematoxylin and eosin-stained slides following the recommendations provided by the International Immuno-Oncology Working Group [27, 38, 39]. The procedure begins by delineating the invasive tumor margin, followed by assessing the density of stromal TILs, calculated as the ratio of immune cells to the total stromal area, excluding regions of necrosis, ductal and lobular carcinoma *in situ*, and normal breast tissue. Although both stromal and intratumoral TILs, which are located within the tumor core, are observed, intratumoral TILs are excluded from standardized reporting in BC based on the recommendations of the International Immuno-Oncology Working Group. The preference for assessing stromal TILs over intratumoral TILs is based on their higher prevalence and variability, which are expected to provide more reliable measurements [40]. However, this manual approach of quantifying TILs presents significant challenges. Firstly, the evaluation performed by pathologists is intrinsically qualitative and subject to interobserver variability [41]. Secondly, hematoxylin and eosin staining oversimplifies the nature of the immune response, as it does not allow for the distinction between different TIL subtypes, each potentially playing different roles within the tumor microenvironment [42]. Thirdly, this method does not estimate the spatial aspects of the TIL distributions that could contain valuable clinical information [43–45]. Furthermore, the omission of intratumoral TILs from the assessment may miss the potential of comprehensive immune microenvironment evaluation [40, 43], especially in cancers where the presence of intratumoral TILs indicates a strong immune response within the tumor core. In response to these limitations, there has been a move toward more granular DIA-based assays that allow more detailed and specific assessment of immune cell subtypes in the tumor microenvironment. In particular, the Immunoscore system [46] offered a robust and standardized way to quantify immune cell density and location – specifically CD3-positive and CD8-positive (CD8+) T-cells – both within the tumor and at its margin. This scoring system, which has been shown to predict patient outcomes more effectively than traditional staging in diseases like colorectal cancer [47], highlights the importance of the immune contexture of the tumor. The computational method of Immunogradient, proposed by Rasmusson et al. [48], enabled precision sampling of the tumor-stroma interface zone (IZ) with quantification of spatial distribution profiles of immune cells across the IZ in several solid tumors, including early ER+ invasive breast carcinoma [48] and HER2-borderline (IHC 2+) BC [49]. The immunogradient indicators consistently performed as independent prognostic markers in the studied patient cohorts.

In this study of 252 patients with ER+HER2– BC and 63 patients with TNBC, we assessed the potential of computational biomarkers of both Ki67-ITH and CD8-immunogradient to predict BCSS in the context of conventional clinical, pathology, and IHC characteristics. We employed DIA for automated quantification of ER, PR, Ki67, and HER2 IHC markers, followed by hexagonal grid-based computing of Ki67-ITH and CD8-immunogradient indicators. Our study demonstrates that both computational biomarkers are independent and can improve prognostic stratification with regard to BCSS.

## Materials and methods

### Patients and clinicopathological characteristics

This retrospective study included 252 patients with ER+HER2– BC and 63 with TNBC selected from an initial cohort of 328 patients with invasive breast carcinoma of no special type. All participants were treated at the National Cancer Institute (Vilnius, Lithuania) between 01/10/2007 and 28/11/2014 and were investigated at the National Center of Pathology, an affiliate of the Vilnius University Hospital Santaros Klinikos (Vilnius, Lithuania). All data was accessed between 09/01/2024 and 05/03/2024 for research purposes. The study excluded patients who had undergone neoadjuvant therapy, were under the age of 35, were diagnosed with distant metastases at the first diagnosis, had incomplete clinicopathologic information, or lacked tumor blocks for additional IHC staining. Subsequently, four cases with low tumor content (< 6 mm^2^ by DIA, representing the 5^th^ percentile for tumor area in the surgical excision samples of the patient cohort) in the CD8 whole-slide images were excluded from further analyses. Clinical and pathological data, including age at the time of surgery, stage at diagnosis, histological grade, pathological tumor size, pathological lymph node status, and surrogate BC subtype, were retrospectively collected from the patient’s medical records. A summary of patient and tumor characteristics is presented in Table 1. Clinical stage was determined following the 8^th^ edition of the American Joint Committee on Cancer [50]. ER and PR positivity was defined as 10% or more of tumor cells exhibiting nuclear staining, as determined by pathology report. HER2 status was determined based on protein overexpression assessed by IHC; cases with an IHC score of 0 or 1+ were considered negative based on the intensity and percentage of IHC staining, and cases with an IHC score of 2+ underwent routine testing by a HER2 fluorescence *in situ* hybridization test to confirm HER2 negativity or positivity. Ki67 proliferation rate was categorized as low (< 20%) or high (≥ 20%) according to [51]. Surrogate intrinsic molecular subtyping of BC was determined using four routine IHC biomarkers: ER, PR, HER2, and Ki67, following the European Society for Medical Oncology clinical practice guidelines [52] with slight modifications. The subtypes were defined as follows: (a) luminal A-like subtype was defined as ER/PR+ and HER2– with a low Ki67 proliferation rate and low/intermediate tumor grade; (b) luminal B-like (HER2–) subtype was defined as ER/PR+ and HER2– with a high Ki67 proliferation rate and/or high-grade morphology; and (c) TNBC was identified by the absence of ER, PR, and HER2. BCSS was selected as the endpoint; survival time was defined from the date of surgical tumor removal until death from BC. Follow-up time for participants was restricted to ten years after the surgery.

**Table 1:** Clinical and pathological characteristics by BC subtype.

BC subtype	ER+HER2– BC group	TNBC group
**Number of patients, n (%)**	252 (100%)	63 (100%)
**Clinicopathological characteristics**		
**Age at the time of surgery, years**		
Mean (±standard deviation)	60.7±12.5	57.3±13.4
Median	62	56
Range	36–88	36–85
**Sex assigned at birth, *n* (%)**		
Female	252 (100%)	63 (100%)
Male	0	0
**Follow-up of breast cancer-specific survival, months**		
Mean (±standard deviation)	106.7±21.6	92.6±38.4
Median	114.8	114.3
Range	8.4–120	12.5–120
Deceased, n (%)	33 (13.1%)	15 (23.8%)
**Stage at diagnosis, n (%)**		
I	96 (38.1%)	19 (30.2%)
II	131 (52.0%)	39 (61.9%)
III	25 (9.9%)	5 (7.9%)
IV	0	0
**Pathological tumor invasion stage (pT), n (%)**		
pT1	145 (57.5%)	27 (42.9%)
pT2	107 (42.5%)	36 (57.1%)
pT3 or pT4	0	0
**Pathological lymph node status (pN), n (%)**		
pN0	148 (58.7%)	48 (76.2%)
pN1	79 (31.4%)	10 (15.9%)
pN2	19 (7.5%)	4 (6.3%)
pN3	6 (2.4%)	1 (1.6%)
**Histological grade (G), n (%)**		
G1	42 (16.7%)	0
G2	142 (56.3%)	4 (6.3%)
G3	68 (27.0%)	59 (93.7%)
**Histological type, n (%)**		
Invasive breast carcinoma of no special type	252 (100%)	63 (100%)
Other types	0	0
**Surrogate intrinsic BC subtype, n (%)**		
Luminal A-like BC	120 (47.6%)	0
Luminal B-like (HER2–) BC	132 (52.4%)	0
Luminal B-like (HER2+) BC	0	0
TNBC	0	63 (100%)

BC: breast cancer; ER+: estrogen receptor-positive; HER2: human epidermal growth factor receptor 2; HER2–: human epidermal growth factor receptor 2-negative; TNBC: triple-negative breast cancer

The study was conducted following the guidelines of the Declaration of Helsinki and was approved by the Lithuanian Bioethics Committee (reference number: 40, approval date: 03/08/2007, approval number: 33). Written informed consent to participate in the study was obtained from each patient before study entry. For subsequent biomarker testing (CD8 and ITH assessment) on previously collected and fully anonymized samples, the Lithuanian Bioethics Committee waived the requirement of individual informed consent (approval update date: 12/09/2017, approval update number: 6B-17-189, and 12/01/2023, approval update number: 6B-23-8).

### Immunohistochemistry

Each formalin-fixed paraffin-embedded block of a surgically excised tumor was used for routine hematoxylin and eosin-stained slides. Subsequently, the slides were reviewed by a pathologist (RBV) to select the most informative tumor block with the highest proportion of invasive tumor tissue for IHC testing. For each case, five full-face paraffin sections were cut at 3 μm thickness and mounted on positively charged slides. Ready-to-use antibodies for ER, PR, HER2 (SP1, 1E2, and 4B5, respectively, Ventana, Tucson, AZ, USA), Ki67 (MIB-1 clone antibody, Dako, Glostrup, Denmark; dilution 1:200), and CD8 (C8/144 B clone antibody, Dako, Glostrup, Denmark; dilution 1:100) were used. IHC staining was performed using the Roche Ventana BenchMark ULTRA automated staining system (Ventana Medical Systems, Oro Valley, AZ, USA), and visualized using the ultraView Universal DAB Detection kit (Ventana Medical Systems, Oro Valley, AZ, USA). Mayer’s hematoxylin was applied for counterstaining. IHC staining and sectioning were performed as described in detail in the protocol available at http://dx.doi.org/10.17504/protocols.io.q26g71qxqgwz/v1, and is included as an S1 File with this article.

### Digital image analysis and calculation of indicators

The IHC slides were scanned at 20× magnification, with a resolution of 0.5 μm per pixel and a numerical aperture of 0.75, using an Aperio AT2 DX Slide Scanner (Leica Aperio Technologies, Vista, CA, USA). DIA of the whole-slide images was performed using HALO AI (version 3.5.3577; Indica Labs, USA). HALO AI DenseNet v2 classifier (version 3.5.3577; Indica Labs, USA) was trained using manual annotations provided by a pathologist (RBV) to segment tumor tissue, stroma, and background, which included necrosis, artifacts, and glass. The HALO AI validation tool revealed that the automated identification of tumor tissue in the whole-slide images achieved an F-score of 0.90242, 0.96294 for stroma, 0.95657 for glass, and 0.98316 for artifact detection. The HALO Multiplex IHC algorithm (version 3.1.4; Indica Labs, USA) was used for segmenting and extracting the coordinates of ER, PR, Ki67, CD8, and HER2 2+ and 3+ positive cells with complete membrane staining and for computing the percentages of ER, PR, and Ki67 positive cells, as well as HER2 2+ and 3+ positive cells within the tumor compartment, as identified by the DIA tissue classifier for each whole-slide image. DIA steps, including whole slide image scanning and tissue segmentation, can be found in the detailed protocol available at http://dx.doi.org/10.17504/protocols.io.q26g71qxqgwz/v1 and is included as an S1 File with this article.

The assessment of the ITH of Ki67 positivity rate was performed by systematic subsampling of the DIA data by a randomly positioned hexagonal grid that covered the entire tissue area, as described previously [20]. Based on DIA-extracted coordinates tumor cells were subsampled by a dense grid of hexagons with a side length of 1050 pixels, corresponding to 262.5 μm. Hexagons with fewer than 50 cells were considered insufficiently sampled and were excluded from further analyses. Subsequently, the percentages of Ki67-positive cells were computed for each hexagon and ranked into 10 intervals: 0%–10%, >10%–20%, >20%–30%, etc. Based on the ranks, a co-occurrence matrix was constructed to compute Haralick’s texture indicators, including homogeneity, entropy, contrast, dissimilarity, and energy [53]. As established in the previous study [19], among these indicators, Haralick’s entropy of Ki67 proliferation index was identified as the most informative prognostic indicator for ITH in the same ER+HER2– BC patient cohort; therefore, this parameter was chosen for this study, along with other IHC and immune response indicators.

**Fig 1:**
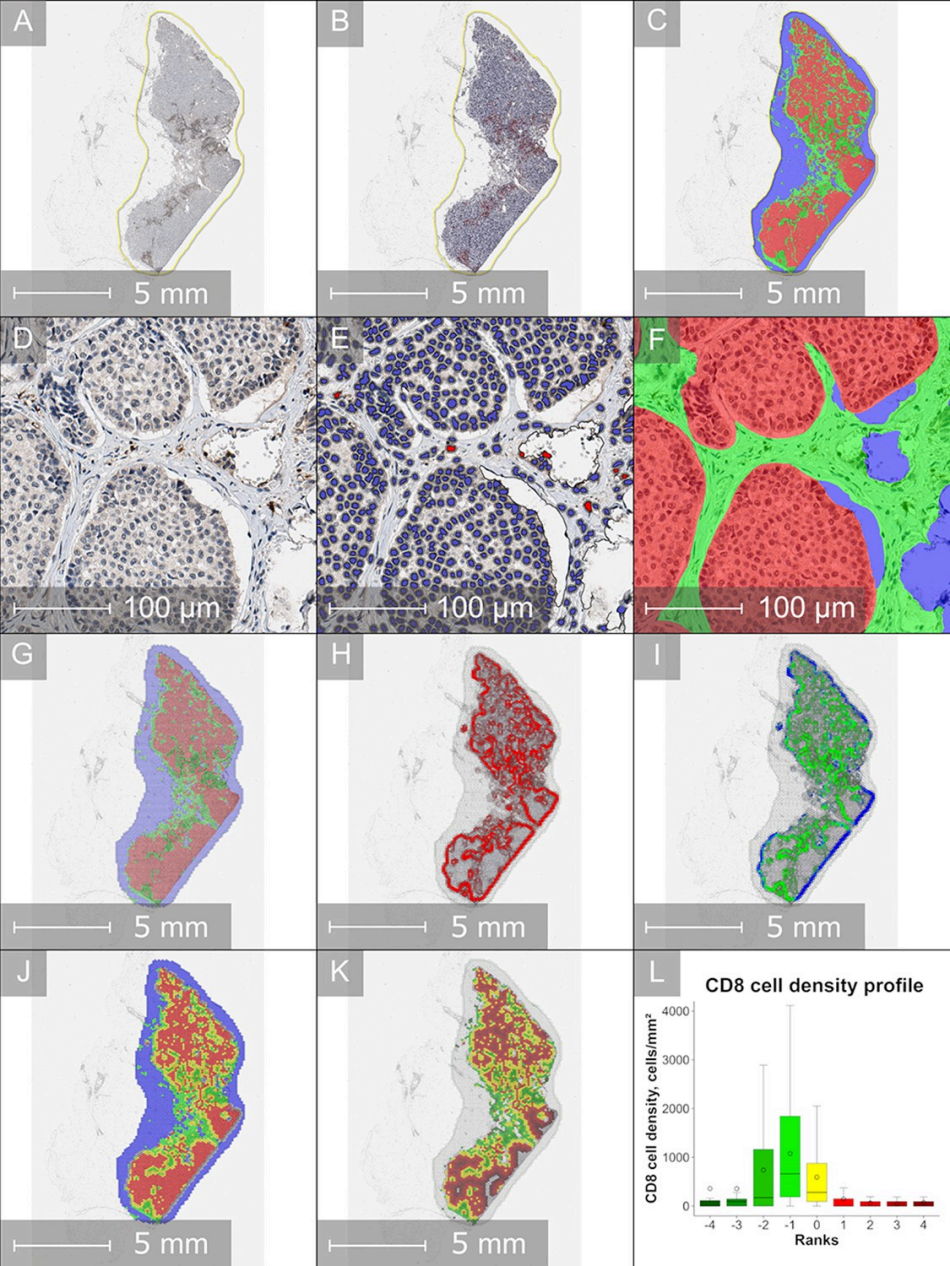
Detection of tumor edge and interface zone to assess CD8+ cell density profiles. (A) Whole-slide image of breast cancer tissue immunohistochemically stained for CD8+ cells, scanned at 20× magnification. CD8+ positive cells are stained brown, while negative cells are stained blue. This image was used for subsequent digital image analysis. (B) Quantitative digital image analysis of CD8 immunohistochemistry, focusing on the whole tumor tissue region (outlined with a yellow border), where CD8+ cells are marked in red and negative cells in blue. (C) Pixelwise segmentation of tumor epithelium versus stroma by HALO AI, with tumor regions shown in red, stroma in green, and background in blue. (D) Magnified view of a selected region from the CD8 immunohistochemistry slide, showing detailed CD8+ cell staining (brown) and tissue morphology. (E) Digital analysis result of CD8+ cell segmentation in the same magnified region, where CD8+ cells are marked in red and negative cells in blue. (F) Pixelwise classification of the magnified view region by HALO AI, with tumor areas shown in red, stroma in green, and background in blue. (G) Hexagonal grid overlay (hexagon side length – 65 μm, equivalent to 260 pixels) applied to systematically subsample the CD8+ digital analysis results. Each hexagon is color-coded based on the proportion of tumor (red), stroma (green), and background (blue) area fractions. (H) Detection of tumor area fractions within each hexagon, showing tumor area variation along the three hexagonal axes. Red hexagons indicate regions with significant tumor area changes. (I) Classification of tumor area changes based on the type of neighboring tissue, distinguishing tumor-stroma (green hexagons) and tumor-background (blue hexagons) transitions. (J) Identification of the tumor edge based on tumor-stroma transitions. Hexagons with abrupt tumor area changes are classified as tumor edge (yellow hexagons, rank 0). Adjacent hexagons within the interface zone are classified as tumor aspect (red) or stroma aspect (green). (K) Extracted interface zone, comprising 9 hexagons in width. Tumor edge hexagons are shown in yellow (rank 0), with adjacent tumor aspect hexagons (ranks 1–4) in red and stroma aspect hexagons (ranks -1 to -4) in green. The intensity of the color reflects the distance from the tumor edge. (L) CD8+ cell density profile across the interface zone, represented by a box-and-whisker plot. The y-axis indicates CD8+ cell density (cells/mm^2^), while the x-axis corresponds to the ranks from -4 to 4 within the interface zone. Stroma aspects (ranks -4 to -1) are shown in green, the tumor edge (rank 0) in yellow, and tumor aspects (ranks 1 to 4) in red. The plot visualizes the mean, median, and variance of CD8+ cell density across these ranks, highlighting differences between the stroma and tumor compartments.

Spatial distribution indicators of CD8+ T-cell positivity with automated tumor edge and IZ extraction were calculated using the computational approach, described in detail by Rasmusson et al. [48]. Briefly, CD8 IHC slides (Fig 1A–1F) were processed by DIA to obtain the tissue classes for each pixel and extract coordinates of CD8+ and CD8-negative cells. DIA data were then assigned to corresponding hexagons based on their coordinates (Fig 1G); hexagons with a diameter of 260 pixels, equivalent to a hexagon side length of 65 μm, were used. The identification of the tumor edge between the stroma and tumor regions is based on abrupt changes in the proportions of tissue class areas across the grid (Fig 1H and 1I). Hexagons located at this edge were assigned a rank of 0 (Fig 1J), and the ranks for the remaining hexagons were assigned based on their hexagonal distance to the tumor edge. To distinguish between the tumor and stroma regions, hexagons within tumor regions were assigned positive ranks, while those on the stroma side received negative ranks. In this study, a nine-hexagon-wide IZ with a central edge of one rank width was selected for subsequent calculations and analysis of CD8+ cell distribution in BC tissue samples (Fig 1K). Immune response indicators were calculated from the IZ to represent the CD8+ cell density patterns (Fig 1L). These indicators included:

1. Mean and standard deviation of CD8+ T-cell density: CD8+ densities and their dispersion were calculated separately for three aspects: tumor, tumor edge, and stroma within the IZ.
2. Immunodrop (ID): to quantify the abrupt drop of immune cell density across the tumor edge, the ID was defined as the ratio of the mean CD8+ density in rank -1 (representing the stroma aspect) and mean CD8+ density in rank 1 (representing the tumor aspect).
3. Center of mass (CM) is a physical concept that for a single line expresses the point of equilibrium for some mass distribution along the line.

### Statistical methods

Statistical analyses and plots were performed using SAS (version 9.4; SAS Institute Inc., Cary, USA) and R software (version 4.1.0). A statistical significance level of *p* < 0.05 was chosen as the threshold. The normality of the distribution of continuous variables was assessed using the Kolmogorov‒Smirnov test, and correlations between variables were assessed using Spearman’s correlation coefficient. A two-sided Welch t-test was employed to assess the homogeneity of variance. Significant associations between CD8+ cell density indicators and categorical parameters (clinicopathological or different tumor regions) were assessed through pairwise comparisons using the Wilcoxon test, and a global comparison of all patient groups was performed using the Kruskal–Wallis test. Factor analysis was performed using principal component analysis as the factoring method, with factors selected based on eigenvalues exceeding 1. Prior to the analysis, Bartlett’s and Kaiser–Meyer–Olkin tests were used to evaluate the suitability of the data. An orthogonal varimax rotation of the initial factors was applied.

The Cutoff Finder tool (Charité University, Berlin, Germany) [54] was used to determine the optimal cutoff values for each indicator and assess their prognostic power for BCSS. Variables with a *p*-value of less than 0.05 in the univariate analysis were selected for further analysis in multivariable Cox regression models. Cox proportional hazards models were fitted by componentwise likelihood-based boosting through the CoxBoost R package. For prognostic modeling, the dataset was split into training and testing subsets as follows: in the ER+HER2– group, 189 patients were in the training set, and 63 patients were in the testing set; in the TNBC group, 47 patients were in the training set, and 16 patients were in the testing set. During the training process, 5-fold cross-validation was applied. After training, each model was evaluated on a hold-out test set, and Harrell’s concordance index (C-index) was used to assess the prediction performance of different initial models. The estimation of BCSS distributions was achieved by the Kaplan–Meier method, and the statistical significance of differences between categorized groups was evaluated using the log-rank test. Additionally, Cox proportional hazard analyses were conducted to examine the independent prognostic significance of the conventional IHC, Ki67-ITH, and CD8+ cell density indicators in the context of clinicopathologic variables. The proportional hazards assumption was tested by examining the time-dependent covariates and the Schoenfeld residuals of the Cox models [55]. The fit of different regression models was assessed by comparing the likelihood ratio (LR). Receiver operating characteristic curve analysis was performed to evaluate the diagnostic performance of the different models. The area under the curve (AUC) was calculated to quantify the overall ability of the models to discriminate between positive and negative outcomes. The AUC values were reported with their corresponding 95% confidence intervals to provide a measure of the precision of the estimates. The statistical significance of the differences between the AUC values of different models was assessed using the DeLong test.

A set of representative cases from both ER+HER2– and TNBC subtypes, illustrating the extracted Ki67-ITH and CD8-immunogradient indicators, is provided as an S2 File with this article.

## Results

### Summary statistics of clinicopathological characteristics, conventional BC IHC, Ki67 heterogeneity, and CD8+ cell density indicators

Patient and tumor characteristics are summarized in Table 1. The mean age of the patients was similar in both groups, ranging from 36 to 88 years. The follow-up period for BCSS varied between 8.4 and 120 months. By the end of the follow-up period, thirty-three and fifteen patients died in the ER+HER2– and TNBC groups, respectively. All patients were diagnosed at the tumor invasion stage 1 or 2. The majority (93.7%) of TNBC cases were represented by histological grade 3.

**Fig 2:**
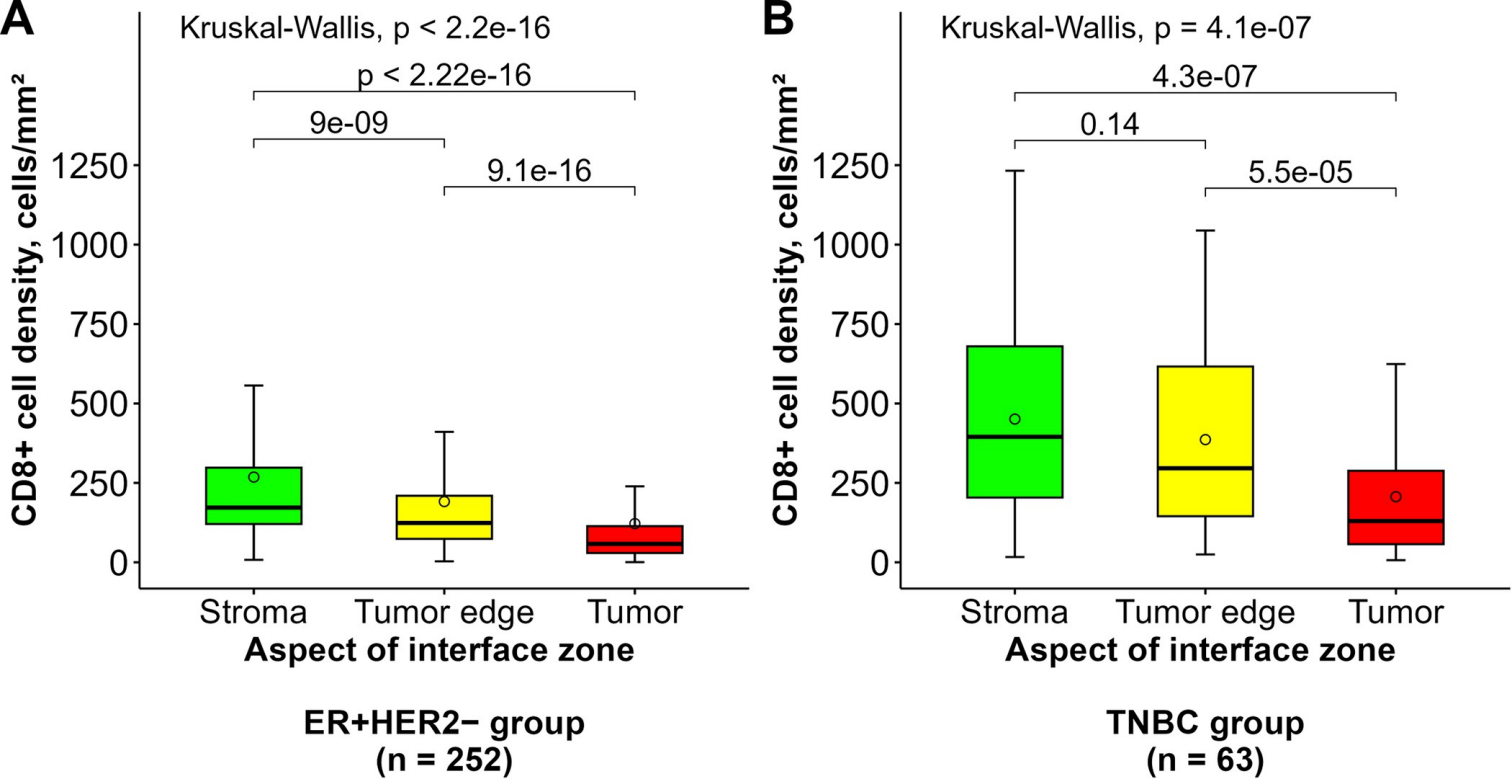
Distribution of CD8+ cell density across interface zone aspects in (A) ER+HER2– and (B) TNBC groups. The box-whisker plots represent CD8+ cell density (cells/mm^2^) across three interface zone aspects: stroma (green), tumor edge (yellow), and tumor (red). In each box, the central line indicates the median CD8+ cell density, while the circle represents the mean. The top and bottom edges of the boxes correspond to the interquartile range, and the whiskers extend to 1.5 times the interquartile range, showing variability outside the quartiles. Outliers beyond this range are not displayed. Statistical significance for pairwise comparisons between different interface zone aspects was assessed using the Wilcoxon test, with *p*-values noted above the connecting horizontal bars. Overall differences across the aspects were analyzed using the Kruskal-Wallis test, with *p*-values provided at the top of each plot. (A) In the ER+HER2– group (n = 252), significant differences were detected across all three zones (stroma, tumor edge, and tumor) as shown by the *p*-values. (B) In the TNBC group (n = 63), significant differences were found between the tumor and stroma, and between the tumor edge and tumor, as indicated by the respective *p*-values. ER+: estrogen receptor-positive, HER2–: human epidermal growth factor receptor 2-negative, TNBC: triple-negative breast cancer.

The relevant summary statistics for the conventional BC IHC, Ki67-ITH, and CD8+ cell density indicators are presented in Table 2. As illustrated in Fig 1, CD8+ cell density profiles were assessed using a hexagonal grid and IZ approach across tumor-stroma transitions, revealing similar CD8+ T-cell distribution patterns in both BC subgroups. In general, the IZ stroma aspect displayed the highest mean of CD8+ cell densities and dispersion, as indicated by the standard deviation values. Meanwhile, the tumor edge demonstrated lower CD8+ cell densities and less dispersion when compared to the IZ stroma aspect in the ER+HER2– BC subgroup (*p* < 0.0001) (Fig 2A); however, this difference did not reach statistical significance in TNBC patients (*p* = 0.14, Fig 2B). The IZ tumor aspect displayed the lowest CD8+ cell densities and lower dispersion in both BC subtypes (*p* < 0.0001). Notably, CD8+ cell densities in all the corresponding IZ aspects were lower in the ER+HER2– BC group than in the TNBC (Table 2).

**Table 2:** Summary statistics of conventional immunohistochemistry, Ki67 heterogeneity, and CD8+ density indicators by breast cancer subtype.

BC subtype	ER+HER2– group (n = 252)			TNBC group (n = 63)			
Indicator	Mean	Standard deviation	Median	Mean	Standard deviation	Median	*p*-value
**Conventional IHC indicators, %**							
**ER**	88.69	16.90	98.15	0.14	0.16	0.06	< 0.0001
**PR**	60.09	38.22	73.17	0.37	0.58	0.14	< 0.0001
**Ki67**	16.28	14.86	12.33	60.14	22.84	65.32	< 0.0001
**HER2** ^**a**^	11.08	16.31	3.56	6.40	13.98	1.26	0.0369
**Ki67-intratumoral heterogeneity**							
**Ki67_entropy**	2.22	1.40	2.36	4.14	1.00	4.29	< 0.0001
**CD8 density indicators, cells/mm** ^**2**^							
**CD8_m_S**	295.77	337.29	173.48	508.21	450.13	406.96	< 0.0001
**CD8_sd_S**	422.41	275.12	336.46	594.47	313.45	539.22	< 0.0001
**CD8_m_TE**	228.13	340.08	126.03	416.53	400.39	298.19	0.0002
**CD8_sd_TE**	299.12	213.03	228.23	454.29	235.58	411.64	< 0.0001
**CD8_m_T**	128.55	238.65	58.11	233.42	315.80	130.88	0.0039
**CD8_sd_T**	169.94	145.88	122.70	252.65	161.22	215.68	0.0001
**CD8_CM**	-0.82	0.72	-0.64	-0.90	0.76	-1.03	0.4471
**CD8_ID**	4.16	4.73	2.74	3.57	3.02	2.92	0.3483

^a^HER2 status was assessed by immunohistochemistry (IHC) and confirmed using a HER2 fluorescence *in situ* hybridization test for cases with an IHC score of 2+; all cases were classified as negative.

BC: breast cancer; ER: estrogen receptor; ER+: estrogen receptor-positive; PR: progesterone receptor; HER2: human epidermal growth factor receptor 2; HER2–: human epidermal growth factor receptor 2-negative; TNBC: triple-negative breast cancer; CD8_m/sd_S/TE/T: CD8+ cell density mean/standard deviation in different interface zone aspects: stroma/tumor edge/tumor; CD8_CM: center of mass of CD8+ cell density; CD8_ID: immunodrop of CD8+ cell density

### Factor analysis of conventional BC IHC, Ki67-ITH, and CD8+ cell density indicators

**Fig 3:**
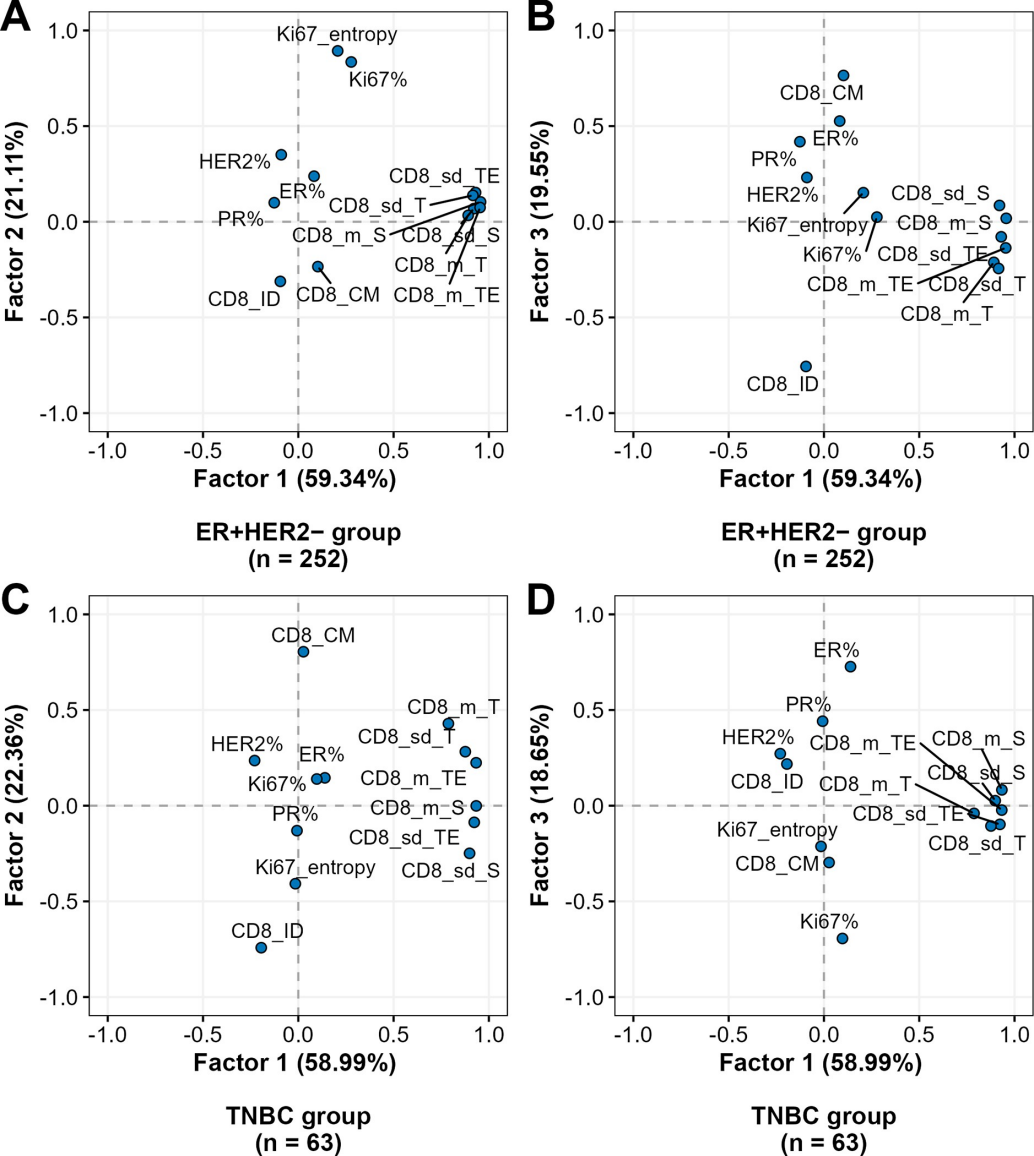
Rotated factor pattern of CD8+ cell density indicators by breast cancer subtype. The factor loadings for factors 1 and 2 in the ER+HER2– and TNBC groups are plotted in (A) and (C), respectively, and those for factors 1 and 3 are plotted in (B) and (D), respectively. Each dot represents an indicator (e.g., the mean or standard deviation of CD8+ cell density in different aspects of the interface zone or the percentage of specific biomarker-positive cells within the tumor compartment). The axes represent the loadings of the indicators on the factors, with higher loadings indicating stronger correlations between the indicator and the factor. This reveals the key associations captured by the factor analysis, with each factor representing a distinct pattern of variability within the dataset. Factors were extracted based on eigenvalues greater than 1 and rotated using an orthogonal varimax rotation for interpretability. BC: breast cancer; CD8_m/sd_S/TE/T: mean/standard deviation of CD8+ cell density in different interface zone aspects: stroma/tumor edge/tumor; CD8_CM: center of mass of CD8+ cell density; CD8_ID: immunodrop of CD8+ cell density; HER2–: human epidermal growth factor receptor 2-negative; ER+: estrogen receptor-positive; TNBC: triple-negative breast cancer.

A factor analysis was performed on the Ki67 expression rates (%) along with the Ki67-ITH and CD8+ cell density indicators, using principal component analysis as the extraction method. Three orthogonally independent factors with eigenvalues greater than 1 were extracted, and an orthogonal varimax rotation was applied to the extracted factors to enhance interpretability by maximizing the independence between them. The results are presented as a scatter plot in Fig 3, where each point represents an individual indicator (e.g., Ki67%, CD8+ cell density in various IZ aspects). Factor 1 was similar in both BC subtypes and was represented by strong positive loadings for variables associated with CD8+ cell density in all IZ aspects. In the ER+HER2– group, factor 3, and in the TNBC group, factor 2 was driven by positive loadings of CM for CD8+ cell density, along with a strong negative loading of the ID indicator. In the ER+HER2– BC subtype, factor 2 was driven by the percentage of Ki67 and its entropy, whereas in the TNBC subtype, factor 3 was characterized by a positive loading for ER% and a negative loading of Ki67%. These three factors collectively explained 69.3% and 64% of the variance in the ER+HER2– and TNBC subtypes, respectively.

### Univariate prognostic value of clinicopathological characteristics, conventional BC IHC, Ki67-ITH, and CD8+ cell spatial density indicators

The univariate prognostic value of conventional BC IHC, Ki67-ITH, CD8+ cell density, and clinicopathological characteristics was evaluated using Kaplan‒Meier analyses, along with hazard ratio (HR) and the log-rank test; the results are summarized in Table 3. In the ER+HER2– group, several factors negatively impacted BCSS, such as grade 3, stage II and III, lymph node involvement, higher Ki67 index, increased entropy of Ki67-positive cancer cell rates, and ID of CD8+ T-cell density. Conversely, higher CD8+ density, its variance within the IZ tumor aspect, and CM of CD8+ cell density were associated with a longer BCSS. For patients diagnosed with TNBC, a statistically significant univariate predictor of worse BCSS was tumor invasion stage 2 and increased entropy of Ki67 expression rate, while higher density and variance in the IZ stroma aspect were associated with a longer BCSS time. No significant stratifications were obtained for patient age at the time of surgery, BC subtype (luminal A-like vs. B-like), or the global expression rates of ER, PR, and HER2 assessed by DIA. Similarly, CD8+ T-cell density and variance within the tumor edge of the IZ did not show statistically significant associations with BCSS.

**Table 3:** Univariate predictors of BCSS by BC subtype.

Univariate regression analysis						
BC subtype	ER+HER2– group (n = 252)			TNBC group (n = 63)		
**Indicator**	**HR**	***p-*value**	**95% CI**	**HR**	***p*–value**	**95% CI**
**Clinicopathological variables**						
**Age (≤ Md vs. > Md)**	1.08	0.7579	0.66–1.78	1.79	0.1600	0.80–4.01
**Grade (G1–2 vs. G3)**	2.07	0.0303	1.08–3.99	N/A^a^	–	–
**Stage (I vs. II-III)**	2.48	0.0310	1.09–5.64	2.30	0.2745	0.52–10.20
**Tumor stage (pT1 vs. pT2)**	1.16	0.6569	0.60–2.22	5.90	0.0195	1.33–26.19
**Lymph node status (pN0 vs. pN1–3)**	2.58	0.0053	1.33–5.01	0.79	0.7079	0.22–2.78
**BC subtype (luminal A-like vs. B-like)**	1.83	0.1653	0.78–4.28	–	–	–
**Conventional IHC indicators by DIA assessment**						
**ER%**	0.55	0.0909	0.27–1.11	0.31	0.0517	0.09–1.08
**PR%**	0.50	0.0517	0.25–1.02	0.43	0.1102	0.15–1.25
**Ki67%**	2.25	0.0191	1.12–4.53	3.74	0.1710	0.49–28.46
**HER2%**	0.51	0.0612	0.27–1.05	0.42	0.0854	0.15–1.17
**Ki67-intratumoral heterogeneity indicator**						
**Ki67_entropy**	2.50	0.0065	1.26–4.94	5.01	0.0006	1.81–13.87
**CD8+ cell density indicators**						
**CD8_m_S**	0.00	0.2096	0.00–Inf	0.14	0.0004	0.04–0.50
**CD8_sd_S**	0.00	0.2350	0.00–Inf	0.33	0.0249	0.12–0.91
**CD8_m_TE**	0.52	0.0618	0.26–1.04	0.00	0.0583	0.00–Inf
**CD8_sd_TE**	0.51	0.0557	0.25–1.03	0.40	0.1426	0.11–1.42
**CD8_m_T**	0.19	0.0002	0.07–0.50	0.22	0.1099	0.03–1.68
**CD8_sd_T**	0.19	0.0006	0.07–0.55	0.21	0.0921	0.03–1.57
**CD8_CM**	0.39	0.0285	0.16–0.93	2.33	0.0920	0.85–6.45
**CD8_ID**	3.30	0.0003	1.67–6.53	0.22	0.1055	0.03–1.66

^a^For the TNBC group, univariate regression analysis for the grade variable (G1–2 vs. G3) was not applicable (N/A) because 93.7% of cases were grade 3 (G3). BCSS: breast cancer-specific survival; BC: breast cancer; ER+: estrogen receptor-positive; HER2–: human epidermal growth factor receptor 2-negative; TNBC: triple-negative breast cancer; HR: hazard ratio; CI: confidence interval; CD8_m/sd_S/TE/T: mean/standard deviation of CD8+ cell density in different interface zone aspects: stroma/tumor edge/tumor; CD8_CM: center of mass of CD8+ cell density; CD8_ID: immunodrop of CD8+ cell density

### Independent predictors of BCSS

All variables significantly associated with the outcome in the univariate analyses (*p* < 0.05, Table 3) were assessed for their independent prognostic value for BCSS by multivariable Cox proportional hazards regression analyses. Five models were generated for each BC subtype, incorporating various sets of variables. Initially, the models included clinical and pathology data alongside conventional BC IHC data, followed by supplementation with Ki67-ITH and immune response data. The details of these models are presented in Table 4.

**Table 4:** Multivariable Cox regression analyses of prognostic factors associated with BCSS.

Multivariable regression analysis									
ER+HER2– group					TNBC group				
Indicator	Hazard ratio	*p*–value	95% CI	χ^2^	Indicator	Hazard ratio	*p*–value	95% CI	χ^2^
**Model 1:** Clinicopathologic and conventional IHC indicators by DIA assessment									
LR: 11.16, *p* = 0.0038, mean Harrell’s C-index: 0.6571, AUC: 0.646 (95% CI: 0.552–0.740)					LR: 7.97, *p* = 0.0047, mean Harrell’s C-index: 0.6884, AUC: 0.694 (95% CI: 0.580–0.808)				
Lymph node status (pN0 vs. pN1–3)	2.42	0.0130	1.21–4.87	6.16	Tumor stage (pT1 vs. pT2)	5.90	0.0195	1.33–26.19	5.46
Ki67%	2.23	0.0246	1.11–4.50	5.05	–	–	–	–	–
**Model 2:** Clinicopathologic and conventional IHC indicators by DIA assessment and Ki67 heterogeneity indicators									
LR: 12.88, *p* = 0.0026, mean Harrell’s C-index: 0.6392, AUC: 0.649 (95% CI: 0.547–0.750)					LR: 16.60, *p* = 0.0002, mean Harrell’s C-index: 0.7168, AUC: 0.794 (95% CI: 0.652–0.935)				
Lymph node status (pN0 vs. pN1–3)	2.62	0.0074	1.29–5.28	7.18	Tumor stage (pT1 vs. pT2)	6.37	0.0157	1.42–28.44	5.84
Ki67_entropy	4.45	0.0055	1.55–12.76	7.71	Ki67_entropy	5.39	0.0013	1.92–15.09	10.28
**Model 3:** Clinicopathologic and CD8+ density indicators									
LR: 22.97, *p* < 0.0001, mean Harrell’s C-index: 0.7251, AUC: 0.722 (95% CI: 0.638–0.806)					LR: 16.79, *p* = 0.0001, mean Harrell’s C-index: 0.8129, AUC: 0.797 (95% CI: 0.656–0.937)				
Lymph node status (pN0 vs. pN1–3)	2.90	0.0034	1.42–5.92	8.60	Tumor stage (pT1 vs. pT2)	4.73	0.0419	1.06–21.14	4.14
CD8_m_T	0.29	0.0237	0.10–0.85	5.12	CD8_m_S	0.17	0.0059	0.05–0.60	7.59
CD8_ID	2.80	0.0071	1.32–5.91	7.25	–	–	–	–	–
**Model 4:** Clinicopathologic, conventional IHC indicators by DIA assessment and CD8+ density indicators									
LR: 28.07, *p* < 0.0001, mean Harrell’s C-index: 0.7096, AUC: 0.751 (95% CI: 0.664–0.837)					LR: 16.79, *p* = 0.0001, mean Harrell’s C-index: 0.8129, AUC: 0.797 (95% CI: 0.656–0.937)				
Lymph node status (pN0 vs. pN1–3)	2.82	0.0043	1.38–5.76	8.14	Tumor stage (pT1 vs. pT2)	4.73	0.0419	1.06–21.14	4.14
Ki67%	2.32	0.0187	1.15–4.67	5.53	CD8_m_S	0.17	0.0059	0.05–0.60	7.59
CD8_m_T	0.28	0.0190	0.10–0.81	5.50	–	–	–	–	–
CD8_ID	2.79	0.0071	1.32–5.90	7.24	–	–	–	–	–
**Model 5:** Clinicopathologic, conventional IHC indicators by DIA assessment, Ki67 heterogeneity, and CD8+ density indicators									
LR: 30.00, *p* < 0.0001, mean Harrell’s C-index: 0.7137, AUC: 0.763 (95% CI: 0.677–0.848)					LR: 16.93, *p* = 0.0002, mean Harrell’s C-index: 0.7887, AUC: 0.806 (95% CI: 0.681–0.932)				
Lymph node status (pN0 vs. pN1–3)	3.30	0.0013	1.60–6.81	10.39	Ki67_entropy	3.31	0.0250	1.16–9.44	5.02
Ki67_entropy	5.95	0.0061	2.01–17.58	10.38	CD8_m_S	0.19	0.0119	0.05–0.69	6.33
CD8_m_T	0.26	0.0056	0.09–0.74	6.27	–	–	–	–	–
CD8_ID	2.93	0.0051	1.38–6.22	7.84	–	–	–	–	–

IHC: immunohistochemistry; DIA: digital image analysis; BCSS: breast cancer-specific survival; BC: breast cancer; HER2–: human epidermal growth factor receptor 2-negative; ER+: estrogen receptor-positive; TNBC: triple-negative breast cancer; HR: hazard ratio; CI: confidence interval; CD8_m_S/T: mean of CD8+ cell density in the interface zone stroma/tumor aspects; CD8_ID: immunodrop of CD8+ cell density

**Fig 4:**
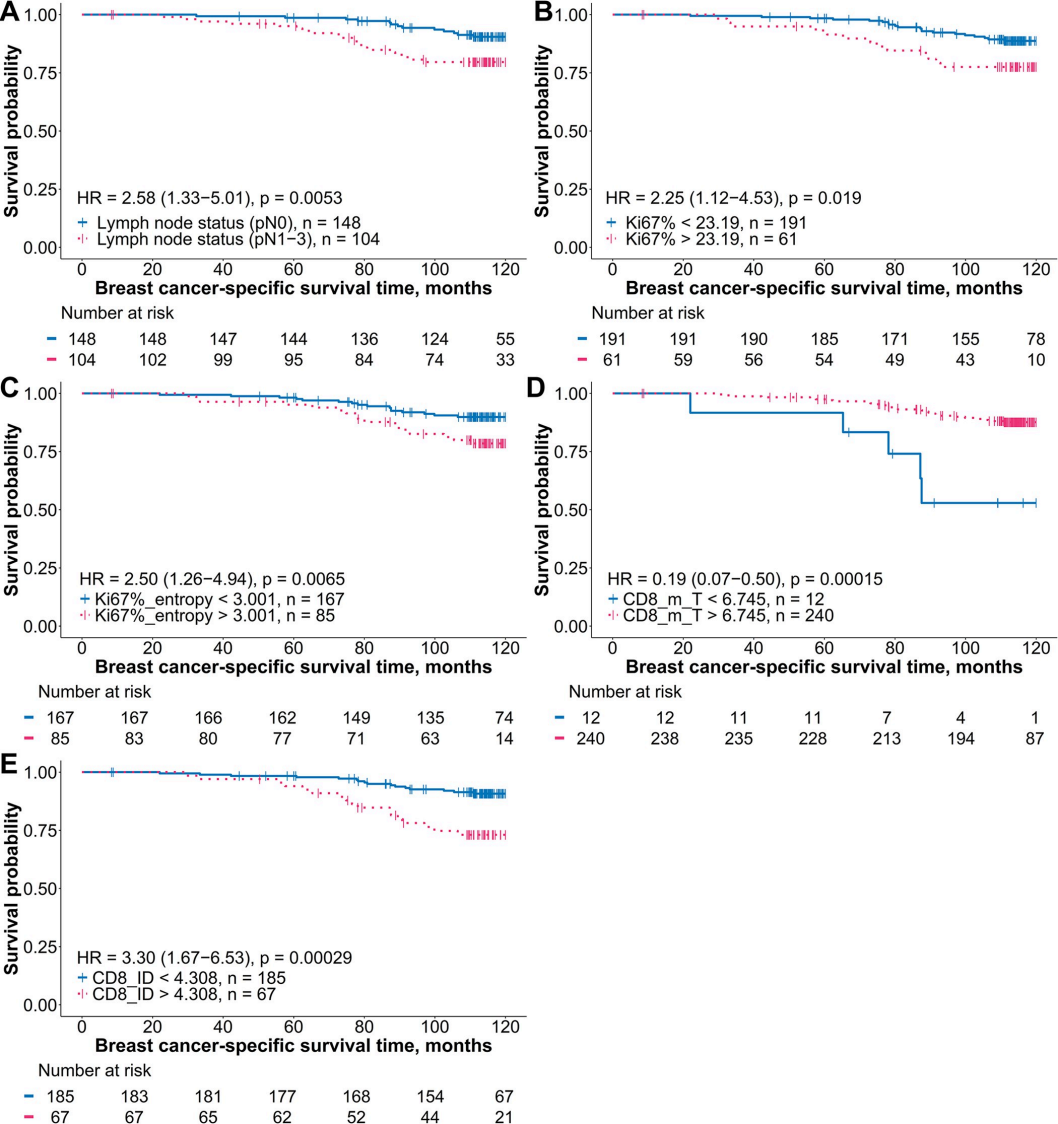
Kaplan–Meier BCSS plots for the independent prognostic indicators in patients with ER+HER2– BC. (A) Survival probability stratified by pathological lymph node status (pN0 vs. pN1–3). (B) Percentage of Ki67-positive cells in tumor tissue. (C) Entropy of local ratios of Ki67-positive cells. (D) Mean of CD8+ cell density in the interface zone tumor aspect. (E) Immunodrop of CD8+ cell density. Kaplan–Meier estimates of breast cancer-specific survival are presented for indicators identified as independent and statistically significant in multivariable Cox proportional hazards analysis. Patients were categorized into two groups for each indicator based on optimal cutoff values determined using the Cutoff Finder [54]. The blue curves represent patients with values below the cutoff, while the pink curves represent those with values above the cutoff. Censored events, indicating patients who were lost to follow-up or who did not experience the event by the end of the study, are marked by vertical tick marks on the survival curves. Statistical differences between groups were evaluated using the log-rank test, and hazard ratios with 95% confidence intervals are displayed within each plot. A table below each plot shows the number of patients at risk at different time points. BCSS: breast cancer-specif survival; DIA: digital image analysis; HER2–: human epidermal growth factor receptor 2-negative; HR: hazard ratio; CD8_m_T: mean of CD8+ cell density in the interface zone tumor aspect; CD8_ID: immunodrop of CD8+ cell density.

In the ER+HER2–BC patients, all models consistently included lymph node status as an independent predictor of worse BCSS. Model 1 (LR: 11.16, AUC: 0.646), utilizing a subset of clinicopathologic characteristics and conventional BC IHC estimates, selected Ki67% as an independent predictor of worse BCSS. The addition of Ki67-ITH (model 2) increased the prognostic power and demonstrated a slightly improved diagnostic performance (LR: 12.88, AUC: 0.649) by substituting Ki67% with the entropy of Ki67%. Model 3 included the CD8+ cell density indicators in combination with the clinicopathologic variables and further improved the prognostic value and diagnostic accuracy (LR: 22.97, AUC: 0.722). It revealed that the ID of CD8+ density was an independent predictor of worse BCSS, while a higher mean of CD8+ density within the IZ tumor aspect was associated with longer BCSS. Model 4 (LR: 28.07, AUC: 0.751) was generated by adding CD8+ cell density profiles to model 1: worse BCSS was associated with higher Ki67%, and ID of CD8+ density, while a longer BCSS was linked to higher CD8+ cell density in the IZ tumor aspect. On the other hand, Ki67% substitution by the Ki67 heterogeneity indicator (Model 5) further increased the LR to 30.00 and the AUC to 0.763 in the ER+HER2– group. It revealed that a shorter BCSS was associated with a higher ITH of Ki67% and ID of CD8+ density, while a longer BCSS was associated with an elevated CD8 density in the IZ tumor aspect. DeLong’s test for comparing the receiver operating characteristic curves indicated significant differences when comparing model 1 with model 4 (*p* = 0.0331) and model 1 with model 5 (*p* = 0.0098), suggesting that models 4 and 5 had significantly better diagnostic performance than model 1. The BCSS probability plots for variables that provided an independent prognostic impact in the ER+HER2– group are presented in Fig 4.

**Fig 5:**
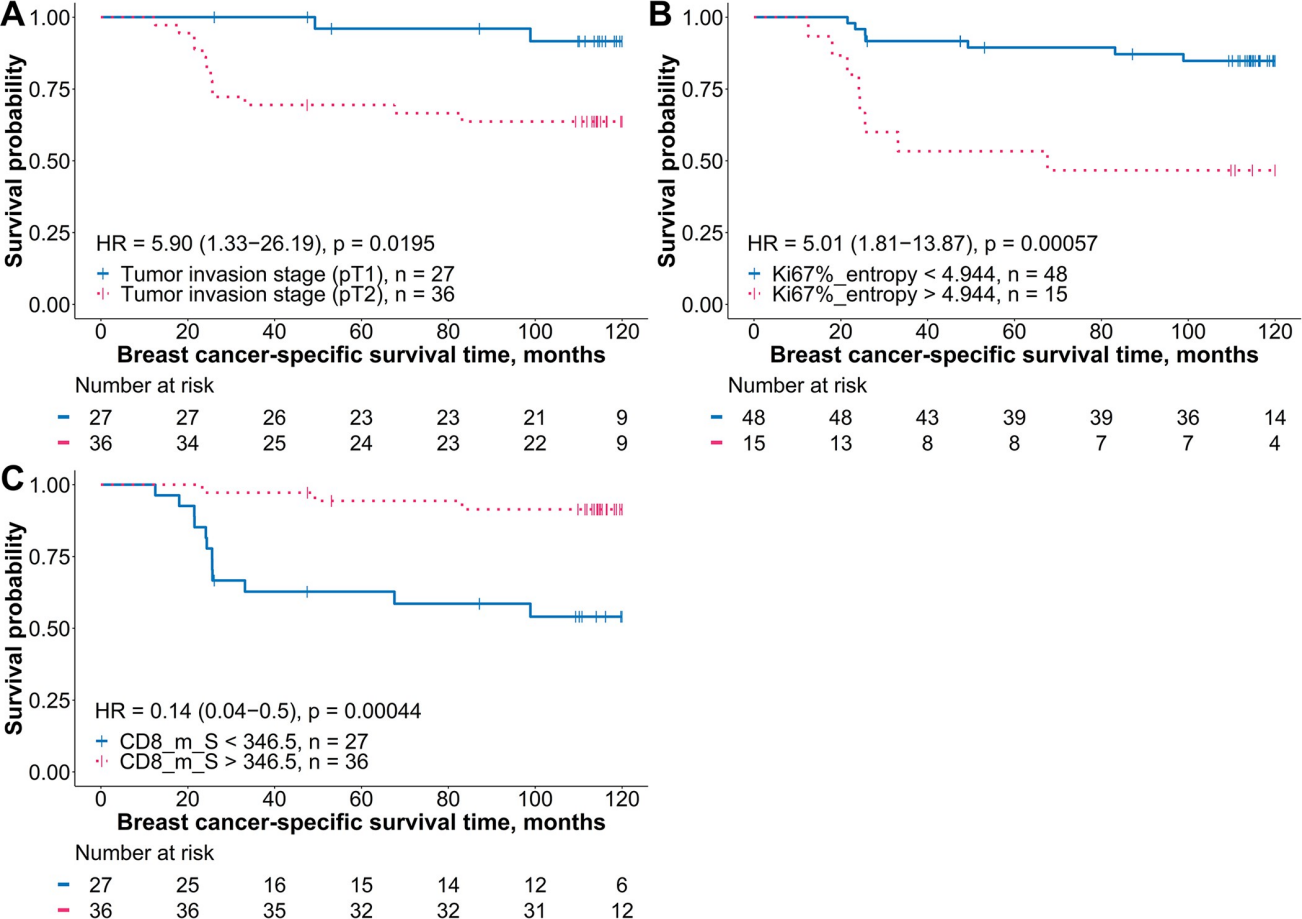
Kaplan–Meier BCSS plots for the independent prognostic indicators in patients with TNBC. (A) Survival probability stratified by pathological tumor invasion stage (pT1 vs. pT2). (B) Entropy of local ratios of Ki67-positive cells. (C) Mean of CD8+ cell density in the interface zone stroma aspect. Kaplan–Meier estimates of breast cancer-specific survival are presented for indicators identified as independent and statistically significant in multivariable Cox proportional hazards analysis. Patients were categorized into two groups for each indicator based on optimal cutoff values determined using the Cutoff Finder [54]. The blue curves represent patients with values below the cutoff, while the pink curves represent those with values above the cutoff. Censored events, indicating patients who were lost to follow-up or who did not experience the event by the end of the study, are marked by vertical tick marks on the survival curves. Statistical differences between groups were evaluated using the log-rank test, and hazard ratios with 95% confidence intervals are displayed within each plot. A table below each plot shows the number of patients at risk at different time points. BCSS: breast cancer-specific survival; TNBC: triple-negative breast cancer; HR: hazard ratio; CD8_m_S: mean of CD8+ cell density in the interface zone stroma aspect.

In contrast, within the TNBC group, model 1 indicated that only the tumor invasion stage 2 was correlated with worse BCSS. Model 2 selected the entropy of Ki67-positive cancer cell rates and tumor invasion status as two independent features associated with worse BCSS. Both, model 3 and model 4, considering CD8+ cell density indicators in combination with clinicopathologic variables and conventional BC IHC indicators, respectively, revealed that tumor invasion stage 2 was associated with worse BCSS, while elevated CD8+ density in the IZ stroma aspect correlated with better BCSS. In model 5, derived from clinicopathologic, conventional IHC, Ki67-ITH, and CD8+ cell density indicators, only Ki67-entropy and CD8+ density in the IZ stroma aspect emerged as independent prognostic indicators; noteworthy, this model did not require any clinicopathological data to predict BCSS. For TNBC, DeLong’s test indicated significant differences when comparing model 1 with model 3 (*p* = 0.0407) and model 1 with model 4 (*p* = 0.0461), suggesting that models 3 and 4 had significantly better diagnostic performance than model 1. The prognostic stratifications based on independent indicators for the TNBC patients are presented in Fig 5.

### Combined prognostic BCSS score

**Fig 6:**
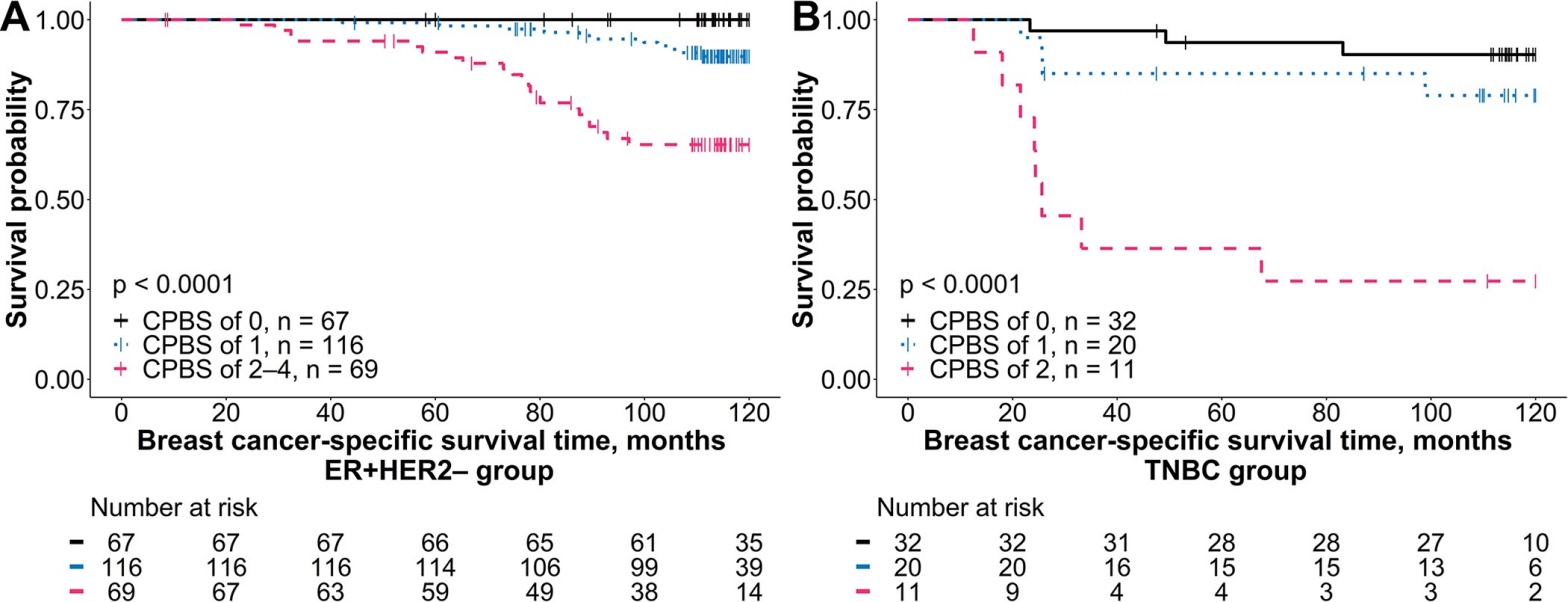
Kaplan-Meier BCSS plot of comprehensive prognostic BCSS score (CPBS) derived from independent predictors. (A) Survival probability stratified by pathological lymph node status, Ki67-entropy, mean of CD8+ cell density in the IZ tumor aspect, and immunodrop of CD8+ cell density in the ER+HER2– BC group. (B) Survival probability stratified by pathological tumor invasion stage, Ki67-entropy, and mean of CD8+ cell density in the IZ stroma aspect in TNBC. A combined prognostic BCSS score was calculated for each breast cancer subtype using independent indicators identified in the best-performing multivariable regression analyses (models 4 and 5, Table 4). In the ER+HER2– group, scores ranged from 0 to 4, while for TNBC, scores ranged from 0 to 3. For the ER+HER2– group, the CPBS was calculated by assigning each indicator a value of 0 (good prognosis) or 1 (bad prognosis) based on its optimal cutoff value, as determined using the Cutoff Finder [54], and then summing these values to derive the final score. This produced three risk groups: low-risk (score of 0, black curve), intermediate-risk (score of 1, blue curve), and high-risk (scores of 2–4, pink curve). Similarly, for the TNBC group, CPBS was calculated by summing the scores of individual indicators to categorize patients into low-risk (score of 0, black curve), intermediate-risk (score of 1, blue curve), and high-risk (score of 2, pink curve). The Kaplan–Meier plots display breast cancer-specific survival (BCSS) curves for each CPBS group. Censored events, indicating patients lost to follow-up or those who did not experience the event by the end of the study, are marked by vertical tick marks on the survival curves. A table below each plot shows the number of patients at risk at different time points. BCSS: breast cancer-specific survival; IZ: interface zone; BC: breast cancer; TNBC: triple-negative breast cancer.

A combined prognostic BCSS score (CPBS) was derived for the ER+HER2– BC and TNBC groups using independent factors identified in the best-performing multivariable regression analyses (models 4 and 5, Table 4). For each indicator, a score of 0 indicated a good prognosis, while a score of 1 indicated a poor prognosis. In the ER+HER2– BC group, the CPBS score was calculated by summing the prognostic categories for lymph node status, Ki67-entropy, mean of CD8+ cell density in the IZ tumor aspect, and ID of CD8+ cell density. For the TNBC group, the CPBS score was derived by summing the prognostic categories for the tumor invasion stage, Ki67-entropy, and mean of CD8+ cell density in the IZ stroma aspect. After calculating the total CPBS score for each patient, they were categorized into three prognostic groups: in the ER+HER2– BC group, a low-risk group had a score of 0, an intermediate-risk group had a score of 1, and a high-risk group had a score of 2–4. In TNBC, the low-risk group included patients with scores of 0 and 1, the intermediate-risk group had a score of 2, and the high-risk group had a score of 3. This scoring system enabled statistically significant risk stratification regarding BCSS (Fig 6). The differences in BCSS probability between the groups were significant: for the ER+HER2– BC subgroup, *p*-values were < 0.0001 for the low vs. high, 0.0096 for the low vs. intermediate, and < 0.0001 for the intermediate vs. high-risk subgroup. In TNBC patients, the results were < 0.0001 for low vs. high, 0.2464 for low vs. intermediate, and 0.0239 for intermediate vs. high-risk.

## Discussion

Our study demonstrates the potential to augment the prognostic stratification of patients with ER+HER2– BC and TNBC by computational assessment of spatial aspects of tumor proliferation and tissue immune response based on DIA data obtained from digitized Ki67 and CD8 IHC slides. In ER+HER2– BC, both a higher CD8+ T-cell density within and its gradient toward the IZ tumor aspect independently contributed to the prediction of improved BCSS. In TNBC, increased CD8+ cell density in the IZ stroma aspect served as an independent feature of better prognosis. The ITH of tumor proliferation represented by Ki67-entropy served as an independent predictor of worse BCSS and was superior to any other Ki67 indicator in both BC subtypes studied. Combining with the impact of lymph node status in ER+HER2– BC and tumor invasion stage in TNBC, prognostic risk assessment could be improved.

In this study, hexagonal grid-based analysis methods reported previously [48], allowed to quantify CD8+ cell density profiles across the tumor-stroma IZ; significant variability and, in general, decreasing density toward the tumor were revealed in both BC subtypes (Fig 2). On the other hand, CD8+ cell densities were significantly (*p* < 0.001) lower in the ER+HER2– BC with lower variance in all IZ aspects and a more pronounced drop of the density at the tumor aspect; one could suggest that this pattern, in general, reflects a lower CD8+ cell response and CD8+ cell penetration into the tumor compartment. These findings are in line with previous studies indicating that TNBC and HER2+ BC are more immunogenic than luminal-like BC [33, 56]. Also, Rapoport et al. [57] and Fortis et al. [58] have reported lower densities of CD8+ cells at the tumor center compared to the invasive margin using DIA in various BC cohorts. Similarly, Jurmeister et al. [59] highlighted considerable variance of immune cell subsets across different tumor compartments in non-small cell lung cancer.

Importantly, in addition to the densities of the immune cell infiltrates within the tumor microenvironment compartments, the immunogradient indicators reflect the directional properties by quantifying the CD8+ cell density profiles across the IZ. In TNBC, we found that the CD8+ T-cell density in the IZ stroma aspect provided the highest independent positive prognostic value, particularly when considered in the context of tumor stage (Table 4, models 3 and 4) and/or Ki67-ITH (Table 4, model 5). Meanwhile, the CD8+ gradient indicators (CM or ID) did not reveal prognostic significance in univariate analyses in TNBC. These findings support the well-established prognostic value of stromal TILs in TNBC outcomes [60–62] and are in line with the recommendation of the International Immuno-Oncology Working [38] that only stromal immune cells should be evaluated for their prognostic value in TNBC.

The prognostic role of stromal CD8+ T cells in TNBC might be explained by the distinct immune landscape of this aggressive BC subtype. While immune exclusion is generally associated with resistance to therapies like immune checkpoint inhibitors [63], stromal CD8+ T cells may still indicate an active immune response that limits tumor progression through interactions with other immune cells, such as macrophages and dendritic cells [64]. This robust stromal immune response, which is more prominent in TNBC due to its higher immunogenicity compared to ER+HER2– BC [33, 56], can restrict tumor growth even without tumor core infiltration, contributing to better survival outcomes in TNBC [60–62]. In contrast, in the ER+HER2– BC, we found that a higher CD8+ cell density at the IZ tumor aspect was positively associated with BCSS. Remarkably, no association with BCSS was found for the CD8+ T-cell densities in the IZ stroma or tumor edge compartments. While the evidence for the prognostic or predictive value of TILs or CD8+ T-cell density in luminal-like BC is limited and controversial [33, 44, 56, 65–68], our findings align with the study by Krijgsman et al. [44], who observed a positive trend between CD8+ density in tumor region and clinical outcomes in a cohort of 236 patients with ER+ invasive BC using machine learning-based approach. They found an independent significant association between longer overall survival and standard deviation of the CD8+ T-cell density within the tumor core. Our study extends these observations by demonstrating the independent prognostic impact of higher CD8+ density in the IZ tumor aspect in the context of pathology indicators (Table 4, model 3) and conventional BC IHC data (Table 4, model 4), and further supplemented by the Ki67-ITH indicator (Table 4, model 5). This suggests that in ER+HER2– BC, immune surveillance within the tumor core is crucial for controlling tumor growth, as these cancers are less immunogenic compared to TNBC. In this context, CD8+ T cells in the tumor core likely have a more direct impact on limiting tumor progression and improving survival, while stromal CD8+ cells have less prognostic relevance due to the generally lower immune activity in this subtype.

In addition to the prognostic value of CD8+ density in the tumor aspect, we report an independent prognostic contribution of the ID indicator of CD8+ density in ER+HER2– BC tumors (Table 4, models 3 and 5), highlighting the value of the density profiles across the tumor-stroma IZ. A more pronounced CD8+ cell density drop (higher ID) across the IZ was associated with worse BCSS. Importantly, neither CD8+ cell densities in any aspect of IZ and nor the spatial CD8 profile indicators (ID and CM) were associated with the variance of any Ki67 indicators, as revealed by factor analyses in both BC types (Fig 3). Notably, CD8+ cell densities in the stroma, tumor edge, and tumor aspects were closely aligned in the factor loadings, reflecting their similar behavior in different regions of the tumor. The factors driven by the spatial CD8 density profiles and the Ki67 indicators were linearly independent while contributing independently to the prognostic models. This highlights distinct biological and clinical contributions of the tumor tissue immune response and proliferation as the hallmarks of cancer. These findings indicate that the prognostic value of CD8+ TILs can be augmented by computationally assessing both their quantity and spatial distribution at the tumor interface. This is particularly important in ER+HER2– BC, where the low CD8+ cell densities and their low dynamic range may require high-precision and high-capacity techniques to reveal their clinical importance. This finding supports our previous findings [48], where in a hormone receptor-positive BC cohort of 102 patients, both a lower ID and a higher CM indicator, reflecting the positive immunogradient of CD8+ T-cells toward the tumor within the IZ, were strongly associated with better overall survival.

Previous studies [19, 20, 23, 49, 69, 70] have highlighted the independent prognostic role of Ki67 and PR-ITH indicators in predicting clinical outcomes, including overall survival, BCSS, or disease-free survival in BC, often exceeding the prognostic value of biomarker expression levels or rates assessed by manual or digital IHC scoring techniques. Recently, we reported that Ki67-ITH is an independent and superior prognostic feature representing proliferation in ER+HER2– BC [19]. The current study extends this finding into TNBC: while the global Ki67 percentage in TNBC was not significantly associated with BCSS in univariate analysis, Ki67-entropy demonstrated significant prognostic value both in univariate analysis (HR = 5.01, *p* = 0.0006) and as an independent prognostic factor (HR = 5.39, *p* = 0.0013) in the context of tumor invasion stage (Table 4, model 2). Further, our study reveals that tumor proliferation and immune response features, represented by spatial Ki67 expression and CD8+ cell density profiles, have independent prognostic value in both ER+HER2– BC and TNBC. While this supports the notion of independent progression of biological hallmarks of cancer [71]; in practical terms, it shows the potential of specific and affordable computational IHC biomarkers to enhance the precision of clinical risk stratification, as illustrated by our prognostic models supplemented with these indicators.

To demonstrate the impact of the multivariable regression models, we combined the independent prognostic features (Table 4, models 4 and 5) to construct a combined BCSS scoring system (CPBS) for patients with ER+HER2– BC and TNBC. This model merged clinicopathological data with Ki67-ITH and immune response features to assess BCSS probability categorizing patients into low-risk, intermediate-risk, and high-risk groups. (Fig 6). For the ER+HER2– BC, the model revealed significant differences in BCSS outcomes between the defined risk groups. Patients in the low-risk category of ER+HER2– BC (n = 67), characterized by no lymph node involvement, low Ki67-ITH, high CD8+ density in the IZ tumor aspect, and low ID of CD8+ cell density, achieved 10-year survival probability of 100%. In contrast, the survival probability for those in the high-risk group (n = 69) was only 65.2%. Similarly, in the TNBC group, the CPBS model also showed significant differences in BCSS outcomes among the risk groups. Patients with tumor invasion stage 2, high Ki67-ITH, and low stromal CD8+ T-cell density had a survival rate of 25.7%. In contrast, those in the low-risk and intermediate-risk groups had survival rates of 98.9% and 67.6%, respectively. These results highlight the value of integrating multiple, IHC-based computational biomarkers into prognostic models, offering a more nuanced risk stratification for clinical decision-making.

Our findings necessitate cautious interpretation due to several limitations. Firstly, our models were developed on a limited number of cases, in particular, TNBC with only 63 cases and a relatively small number of events, which may affect the robustness and general applicability of the results. Secondly, the study was conducted in a single center, which may limit the generalizability of our findings to other settings. Further studies are needed in cohorts from multiple centers. Thirdly, the lack of comprehensive data on therapies administered to patients represents a significant limitation, as variations in treatment regimens may impact survival outcomes in BC. While all patients in our cohort received standard treatments – such as tamoxifen for hormone receptor-positive disease and radiotherapy and/or chemotherapy post-surgery – none received HER2-targeted therapies or immunotherapy. However, detailed information on treatment regimens, such as dosages and treatment durations, was unavailable. This limitation introduces potential bias, as patients with similar biomarker profiles may have responded differently to treatment. This highlights the need for future studies with more comprehensive and consistent treatment data to fully assess the prognostic and/or predictive potential of these biomarkers. Nevertheless, our study reveals the potential clinical value of the computational IHC biomarkers, obtained by explicit statistical processing of DIA data.

## Conclusions

We report that the ITH of Ki67 provides an independent prognostic value superior to that of the Ki67 index in both the ER+HER2– and TNBC subtypes. The tissue immune response, represented by spatial CD8+ cell density profiles, was another independent predictor: better BCSS was associated with the incidence of CD8+ cells in the IZ tumor or stroma aspect in ER+HER2– BC and TNBC patients, respectively. The inclusion of both Ki67-ITH and CD8+ density indicators improved the prognostic stratification of the patients.

## Supporting information

S1 File Protocol from protocols.io in pdf format. (PDF)

S2 File Representative cases from both ER+HER2– and TNBC subtypes, illustrating the extracted Ki67-ITH and CD8-immunogradient indicators in pdf format. (PDF)
