# Supplementary material for: Spatial distributions of CD8 and Ki67 cells in the tumor microenvironment independently predict breast cancer-specific survival in patients with ER+HER2– and triple-negative breast carcinoma
Source: PLoS One. 2024 Nov 22;19(11):e0314364. doi: 10.1371/journal.pone.0314364 (PMC11584100; doi:10.1371/journal.pone.0314364)
Supplement: S1 File — (PDF) [file pone.0314364.s001.pdf]

Formalin-fixed paraffin-embedded sample sectioning and immunohistochemistry staining for ER, PR, Ki67, HER2, and CD8 at the National Centre of Pathology, affiliate of Vilnius University Hospital Santaros Klinikos (Vilnius, Lithuania)

RESERVED DOI:
10.17504/protocols.io.q26g71qxqgwz/v1

Aida Laurinavieciene[1,2], Dovile Zilenaite-Petrulaitiene[1,2,3]

[1]Department of Pathology and Forensic Medicine, Institute of Biomedical Sciences, Faculty of Medicine, Vilnius University, Vilnius, Lithuania;
[2]National Centre of Pathology, affiliate of Vilnius University Hospital Santaros Klinikos, Vilnius, Lithuania;
[3]Institute of Informatics, Faculty of Mathematics and Informatics, Vilnius University, Vilnius, Lithuania

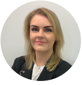

Dovile Zilenaite-Petrulaitiene
National Centre of Pathology, affiliate of Vilnius Universit...

Protocol Info: Aida Laurinavieciene, Dovile Zilenaite-Petrulaitiene . Formalin-fixed paraffin-embedded sample sectioning and immunohistochemistry staining for ER, PR, Ki67, HER2, and CD8 at the National Centre of Pathology, affiliate of Vilnius University Hospital Santaros Klinikos (Vilnius, Lithuania). **protocols.io** **https://protocols.io/view/formalin-fixed-paraffin-embedded-sample-sectioning-dpxf5pjn**

Created: October 18, 2024

Last Modified: October 21, 2024

Protocol Integer ID: 110279

Keywords: formalin-fixed paraffin-embedded, paraffin section, HER2, Ki67, CD8, ER, PR, immunohistochemistry, digital image analysis

Disclaimer

DISCLAIMER – FOR INFORMATIONAL PURPOSES ONLY; USE AT YOUR OWN RISK

The protocol content here is for informational purposes only and does not constitute legal, medical, clinical, or safety advice, or otherwise; content added to **protocols.io** is not peer reviewed and may not have undergone a formal approval of any kind. Information presented in this protocol should not substitute for independent professional judgment, advice, diagnosis, or treatment. Any action you take or refrain from taking using or relying upon the information presented here is strictly at your own risk. You agree that neither the Company nor any of the authors, contributors, administrators, or anyone else associated with **protocols.io**, can be held responsible for your use of the information contained in or linked to this protocol or any of our Sites/Apps and Services.

Abstract

This document describes the procedures for microtomy and immunohistochemistry (IHC) staining of formalin-fixed paraffin-embedded (FFPE) specimens at the National Centre of Pathology, affiliate of Vilnius University Hospital Santaros Klinikos (Vilnius, Lithuania). The protocol focuses on the preparation and sectioning of FFPE tissue samples, followed by IHC staining for key biomarkers including estrogen and progesterone receptors (ER, PR), Ki67, human epidermal growth factor receptor 2 (HER2), and CD8. The document also outlines the steps for performing digital image analysis using HALO (Indica Labs, USA).

Materials

Equipment and instruments:

1. Microtome (HistoCore MULTICUT, Leica Biosystems, Germany)

2. Water bath (Tissue Float Bath Model 1052, GFL Gesellschaft für Labortechnik GmbH, Germany)
3. Cooling plate (HistoCore Arcadia C, Leica Microsystems, Germany)
4. Microtome blade (S35, Feather, Japan)
5. Forceps or fine brush
6. Positively charged glass microscope Slides (TOMO, Matsunami Glass Industry Co., Japan)
7. Slide rack
8. Slide labels (Ventana Medical Systems, USA)
9. Roche Ventana BenchMark ULTRA automated staining system (Ventana Medical Systems, USA)
10. Dry oven (set at 37°C)
11. Aperio AT2 DX slide scanner (Leica Aperio Technologies, USA)

Reagents and Chemicals:

1. Distilled water
2. Antigen retrieval solution (CC1, pH 8.0, Ventana Medical Systems, USA)
3. Primary antibodies: ER (SP1 clone, Ventana Medical Systems, USA), PR (1E2 clone, Ventana Medical Systems, USA), HER2 (4B5 clone, Ventana Medical Systems, USA), Ki67 (MIB-1 clone, Dako, Denmark), CD8 (C8/144 B clone, Dako, Denmark)
4. UltraView Universal DAB Detection Kit (Ventana Medical Systems, USA)
5. Bluing reagent (Biognost, Zagreb, Croatia)
6. 96% ethanol (CAS number: 64-17-5)
7. Isopropanol (CAS number: 67-63-0)
8. Xylene (CAS number: 1330-20-7)
9. Mounting media (Biomount, Croatia)
10. Cover glass (Epredia, USA)

Software and Analytical Tools:

1. HALO AI (version 3.5.3577) (Indica Labs, USA)
2. HALO Multiplex IHC algorithm (version 3.1.4) (Indica Labs, USA)
3. HALO AI DenseNet v2 classifier (version 3.5.3577) (Indica Labs, USA)

Formalin-fixed paraffin-embedded (FFPE) sample microtomy sectioning

1. Wipe the microtome (HistoCore MULTICUT, Leica Biosystems, Germany), the water bath (Tissue Float Bath Model 1052, GFL Gesellschaft für Labortechnik GmbH, Germany), and all work surfaces.

2. Set up the microtome and water bath. Fill the water bath with distilled water and adjust the temperature to 46°C.

3. Place the selected FFPE tissue block on a 4°C cooling plate (HistoCore Arcadia C, Leica Microsystems, Germany) with the sample surface facing down for 5–10 minutes.

4. Insert a new blade (S35, Feather, Japan) into the microtome.

5. Clamp the FFPE block into the microtome holder, aligning the block at a 90° angle to the blade.

6. Trim the block by cutting sections at 10 µm thickness until the tissue surface is fully exposed.

7. Remove paraffin debris from the block and microtome using a dedicated brush.

8. Cut an additional section at 3 µm thickness to further smooth the tissue surface.

9. Cut continuous ribbon sections by smoothly advancing the microtome blade.

10. Carefully guide the ribbon with forceps or a fine brush to prevent folding or tearing.

11. Transfer the ribbon to a water bath set at 46°C.

12. Stretch the paraffin ribbon across the water bath, allowing it to float until wrinkles disappear.

13. Visually inspect the section for integrity.

14 Using a fine brush, gently lift one section from the water bath and carefully place it on a positively charged glass microscope slide (TOMO, Matsunami Glass Industry Co., Japan).

15 Place the slide vertically in a rack to drain excess water.

16 Affix pre-printed, highly resistant slide labels (Ventana Medical Systems, USA) for the specific IHC staining (ER, PR, Ki67, HER2, CD8) to each slide.

17 Repeat the process for additional sections as needed.

18 After collecting the tissue section on the slide, clean the water bath using a paper sheet to remove any residual tissue.

19 Include a positive tissue control section on each slide as the test tissue to ensure proper staining performance.

Immunohistochemistry (IHC) staining of slides using the Roche Ventana BenchMark ULTRA automated staining system

20 Preheat a dry oven to 60°C.

21 Load the slides into a vertical slide rack.

22 Dry the slides at 60°C for 25 minutes.

23 Place the dried slides into the designated slots of the Roche Ventana BenchMark ULTRA system (Ventana Medical Systems, USA).

24 Select the appropriate staining protocol for the specific primary antibody (ER, PR, Ki67, HER2, CD8) on the Roche Ventana BenchMark ULTRA interface.

25 The system automatically performs deparaffinization and antigen retrieval using CC1 solution (pH
8.0, Ventana Medical Systems, USA). Antigen retrieval is conducted for 56 minutes at 95°C for PR and HER2, and for 172 minutes at 95°C for ER, Ki67, and CD8.

26 For ER, PR, and HER2 IHC assays, the system uses ready-to-use primary rabbit monoclonal antibodies (SP1, 1E2, and 4B5 clones, respectively, Ventana, USA) for 16 minutes at 36°C, followed by detection using the ultraView Universal DAB Detection kit (Ventana, USA). For Ki67 and CD8 IHC assays, primary mouse monoclonal antibodies (Ki67: MIB-1, CD8: C8/144 B) are used at 1:200 and 1:50 concentrations, respectively, for 32 minutes at 37°C, followed by detection with the ultraView Universal DAB Detection kit (Ventana, USA).

27 The system counterstains the tissue with Mayer's hematoxylin for 4 minutes, followed by a 4-minute bluing reagent treatment (Biognost, Zagreb, Croatia).

28 After staining, remove the slides from the automated system and place them in a slide rack.

29 For the mounting procedure:

- Submerge the slide rack in fresh distilled water, dunking 3–5 times.
- Submerge slides in 96% ethanol, repeating twice for 2 minutes at room temperature.
- Submerge slides in isopropanol, repeating twice for 2 minutes at room temperature.
- Submerge slides in xylene, repeating twice for 2 minutes.
- Allow the slides to dry at room temperature for 5 minutes.

30 Apply 2–4 drops of mounting media (Biomount, Croatia) to each slide and coverslip with a cover glass (Epredia, USA). Remove any air bubbles by gently applying pressure to the coverslip.

31 Allow the slides to dry for at least 2 hours before imaging.

Digital Image Analysis in Halo (Indica Labs, USA)

32 Scan the slides at 20x magnification using the Aperio AT2 DX Slide Scanner (Leica Aperio Technologies, USA).

33 Perform digital image analysis using HALO AI (version 3.5.3577; Indica Labs, USA).

34 Manually segment the tissue into tumor tissue, stroma, and background to create annotations for the HALO AI DenseNet v2 classifier (version 3.5.3577).

35 Train the HALO AI DenseNet v2 classifier using the manually segmented annotations.

36 Segment the tissue into tumor tissue, stroma, and background, ensuring that regions like necrosis, artifacts, and glass are also classified.

37 Select the appropriate settings for positive and negative cell segmentation using the HALO Multiplex IHC algorithm (version 3.1.4; Indica Labs, USA) to detect and quantify stained cells.
