# Supplementary material for: Spatial distributions of CD8 and Ki67 cells in the tumor microenvironment independently predict breast cancer-specific survival in patients with ER+HER2– and triple-negative breast carcinoma
Source: PLoS One. 2024 Nov 22;19(11):e0314364. doi: 10.1371/journal.pone.0314364 (PMC11584100; doi:10.1371/journal.pone.0314364)
Supplement: S2 File — (PDF) [file pone.0314364.s002.pdf]

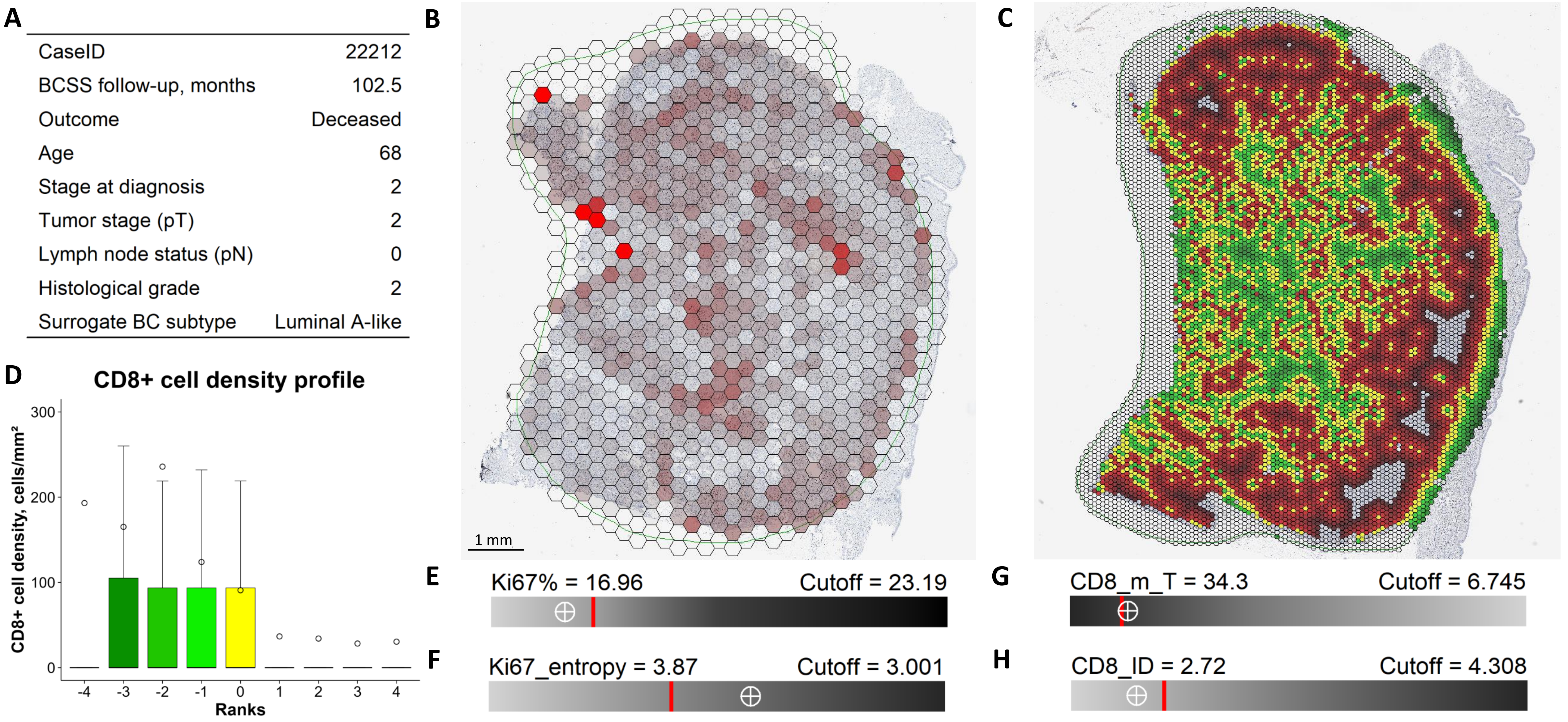

S1 Fig. Extracted Ki67-intratumoral heterogeneity and CD8-immunogradient indicators in breast cancer (BC) case. (A) Clinicopathological indicators for a BC patient. (B) Whole-slide image of BC tissue stained for Ki67, with a hexagonal grid overlaid. Ki67-positive cell percentages within each hexagon are color-coded in red shades to show regions of higher positivity and spatial heterogeneity. (C) Whole-slide image of BC tissue stained for CD8+ cells, with a hexagonal grid and an interface zone (IZ) of 9 hexagons in width extracted. Tumor edge hexagons are yellow (rank 0), tumor aspect hexagons (ranks 1–4) are red, and stroma aspect hexagons (ranks -1 to -4) are green. Color intensity reflects distance from the tumor edge, with rank colors matching those in panel D. (D) Box-and-whisker plot showing CD8+ cell density (cells/mm²) across the IZ, with ranks from -4 to 4 on the x-axis. (E, F, G, H) Ki67 percentage, Ki67 entropy, CD8 mean in the tumor aspect of the IZ (CD8_m_T), and CD8 immunodrop (CD8_ID) values are marked by white circle-cross symbols. The red vertical line marks prognostic cutoff values. Gray shading of bars indicates prognosis, with brighter shades indicating better prognosis and darker shades indicating worse. BCSS: breast cancer-specific survival.

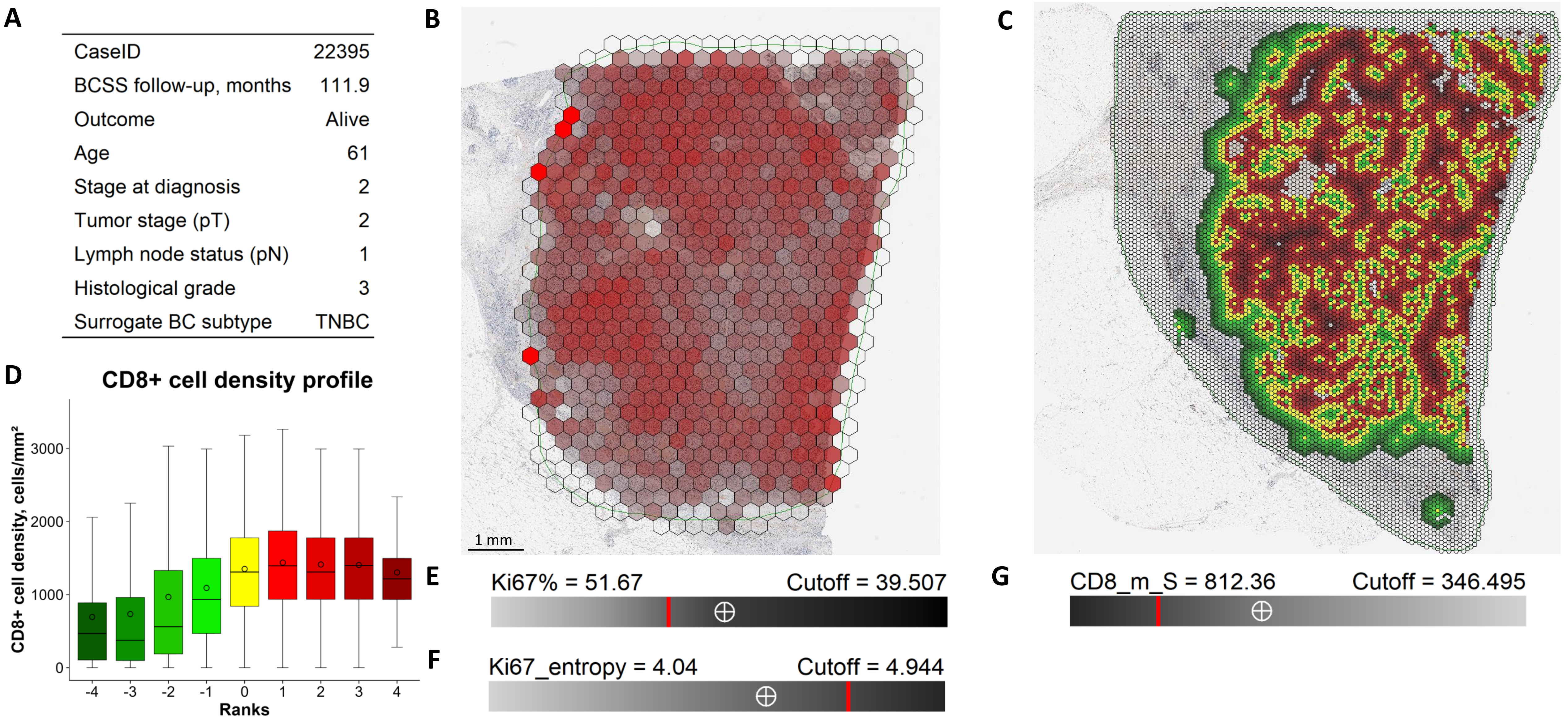

S2 Fig. Extracted Ki67-intratumoral heterogeneity and CD8-immunogradient indicators in breast cancer (BC) case. (A) Clinicopathological indicators for a BC patient. (B) Whole-slide image of BC tissue stained for Ki67, with a hexagonal grid overlaid. Ki67-positive cell percentages within each hexagon are color-coded in red shades to show regions of higher positivity and spatial heterogeneity. (C) Whole-slide image of BC tissue stained for CD8+ cells, with a hexagonal grid and an interface zone (IZ) of 9 hexagons in width extracted. Tumor edge hexagons are yellow (rank 0), tumor aspect hexagons (ranks 1–4) are red, and stroma aspect hexagons (ranks -1 to -4) are green. Color intensity reflects distance from the tumor edge, with rank colors matching those in panel D. (D) Box-and-whisker plot showing CD8+ cell density (cells/mm²) across the IZ, with ranks from -4 to 4 on the x-axis. (E, F, G) Ki67 percentage, Ki67 entropy, and CD8 mean in the stroma aspect of the IZ (CD8_m_S) values are marked by white circle-cross symbols. The red vertical line marks prognostic cutoff values. Gray shading of bars indicates prognosis, with brighter shades indicating better prognosis and darker shades indicating worse. BCSS: breast cancer-specific survival, TNBC: triple-negative breast cancer.

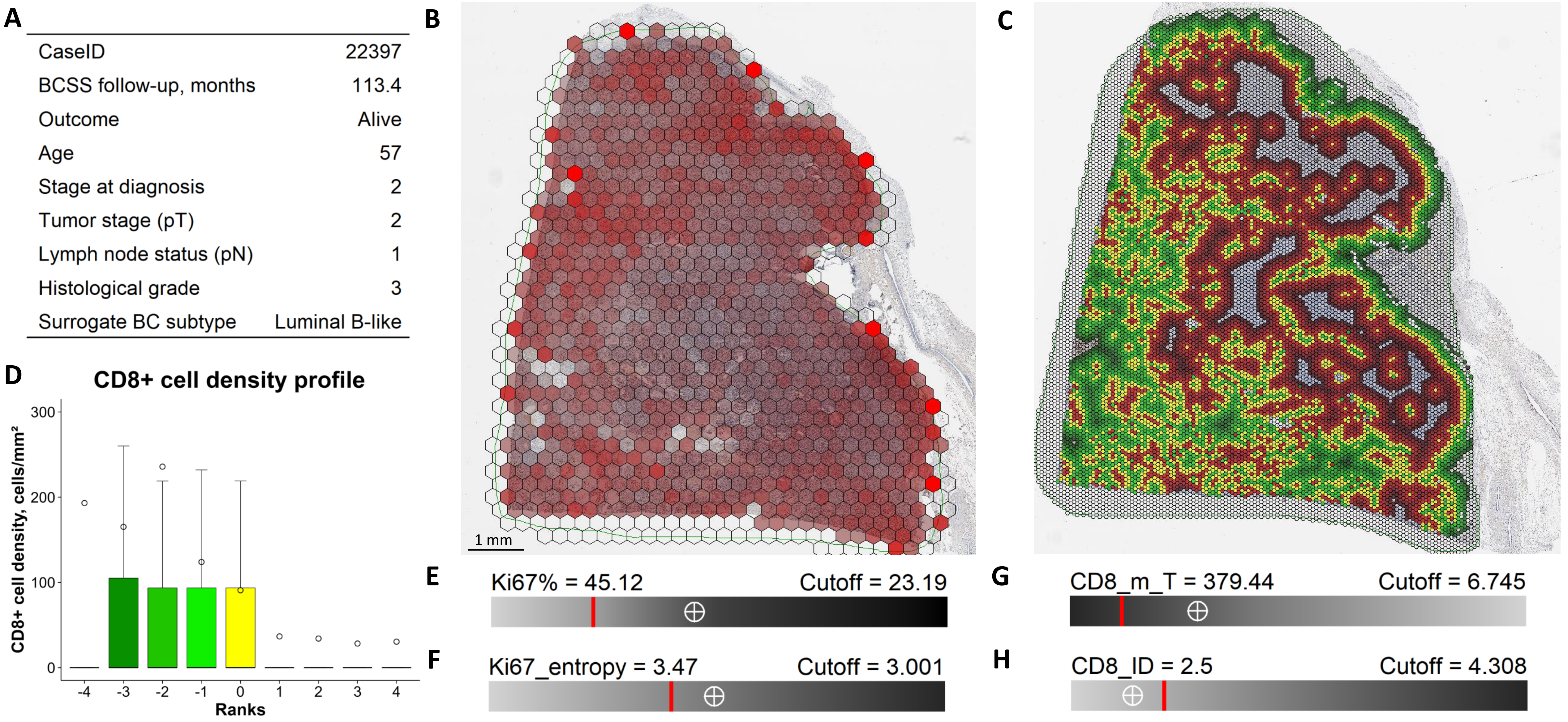

S3 Fig. Extracted Ki67-intratumoral heterogeneity and CD8-immunogradient indicators in breast cancer (BC) case. (A) Clinicopathological indicators for a BC patient. (B) Whole-slide image of BC tissue stained for Ki67, with a hexagonal grid overlaid. Ki67-positive cell percentages within each hexagon are color-coded in red shades to show regions of higher positivity and spatial heterogeneity. (C) Whole-slide image of BC tissue stained for CD8+ cells, with a hexagonal grid and an interface zone (IZ) of 9 hexagons in width extracted. Tumor edge hexagons are yellow (rank 0), tumor aspect hexagons (ranks 1–4) are red, and stroma aspect hexagons (ranks -1 to -4) are green. Color intensity reflects distance from the tumor edge, with rank colors matching those in panel D. (D) Box-and-whisker plot showing CD8+ cell density (cells/mm²) across the IZ, with ranks from -4 to 4 on the x-axis. (E, F, G, H) Ki67 percentage, Ki67 entropy, CD8 mean in the tumor aspect of the IZ (CD8_m_T), and CD8 immunodrop (CD8_ID) values are marked by white circle-cross symbols. The red vertical line marks prognostic cutoff values. Gray shading of bars indicates prognosis, with brighter shades indicating better prognosis and darker shades indicating worse. BCSS: breast cancer-specific survival.

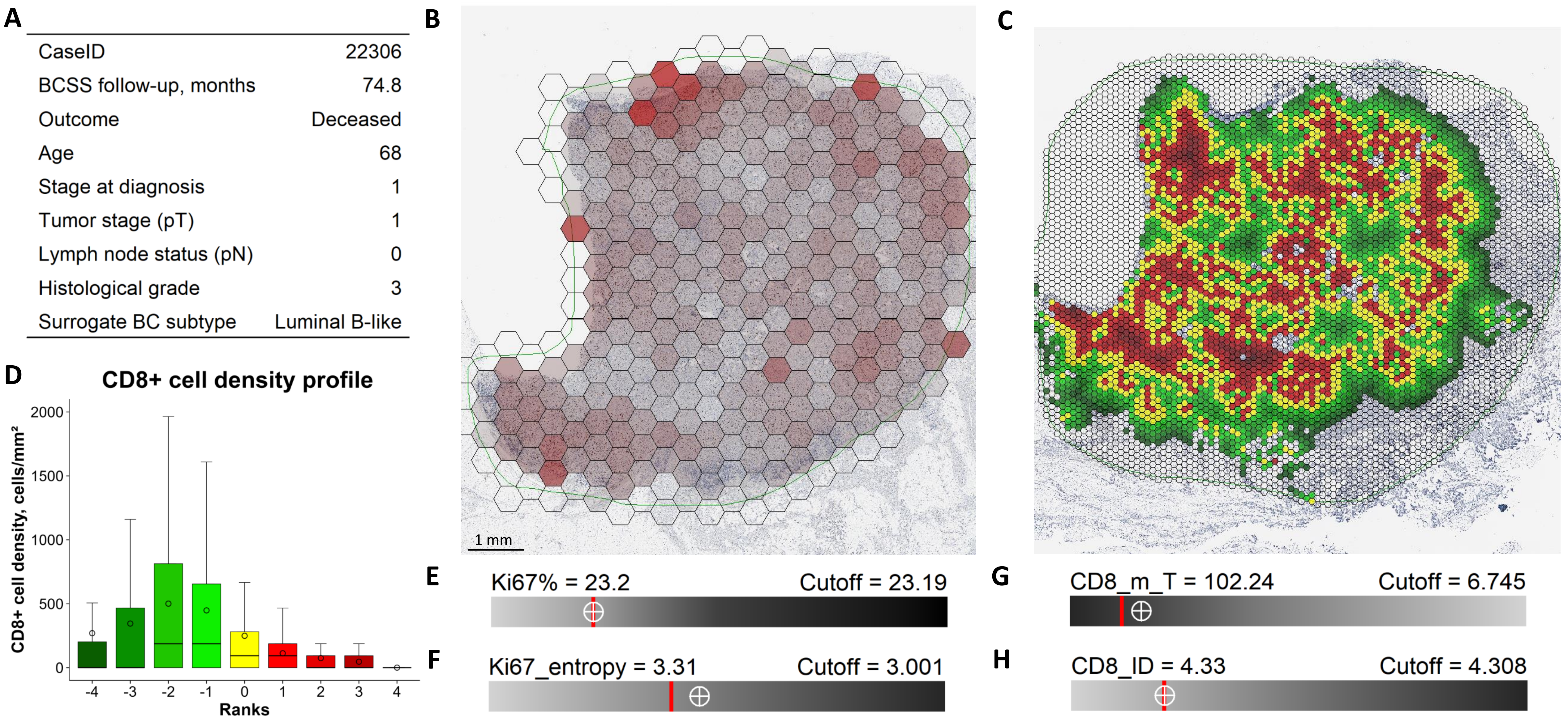

S4 Fig. Extracted Ki67-intratumoral heterogeneity and CD8-immunogradient indicators in breast cancer (BC) case. (A) Clinicopathological indicators for a BC patient. (B) Whole-slide image of BC tissue stained for Ki67, with a hexagonal grid overlaid. Ki67-positive cell percentages within each hexagon are color-coded in red shades to show regions of higher positivity and spatial heterogeneity. (C) Whole-slide image of BC tissue stained for CD8+ cells, with a hexagonal grid and an interface zone (IZ) of 9 hexagons in width extracted. Tumor edge hexagons are yellow (rank 0), tumor aspect hexagons (ranks 1–4) are red, and stroma aspect hexagons (ranks -1 to -4) are green. Color intensity reflects distance from the tumor edge, with rank colors matching those in panel D. (D) Box-and-whisker plot showing CD8+ cell density (cells/mm²) across the IZ, with ranks from -4 to 4 on the x-axis. (E, F, G, H) Ki67 percentage, Ki67 entropy, CD8 mean in the tumor aspect of the IZ (CD8_m_T), and CD8 immunodrop (CD8_ID) values are marked by white circle-cross symbols. The red vertical line marks prognostic cutoff values. Gray shading of bars indicates prognosis, with brighter shades indicating better prognosis and darker shades indicating worse. BCSS: breast cancer-specific survival.

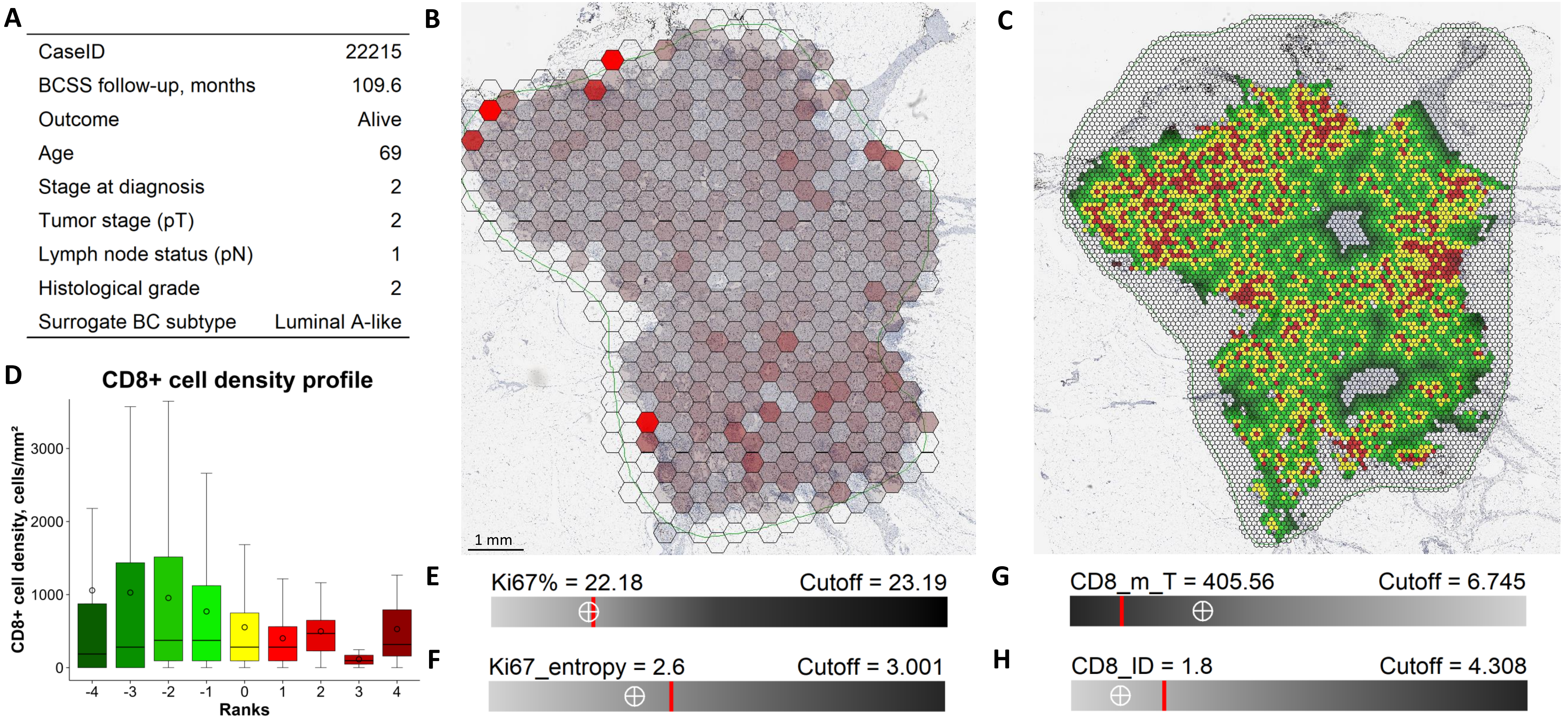

S5 Fig. Extracted Ki67-intratumoral heterogeneity and CD8-immunogradient indicators in breast cancer (BC) case. (A) Clinicopathological indicators for a BC patient. (B) Whole-slide image of BC tissue stained for Ki67, with a hexagonal grid overlaid. Ki67-positive cell percentages within each hexagon are color-coded in red shades to show regions of higher positivity and spatial heterogeneity. (C) Whole-slide image of BC tissue stained for CD8+ cells, with a hexagonal grid and an interface zone (IZ) of 9 hexagons in width extracted. Tumor edge hexagons are yellow (rank 0), tumor aspect hexagons (ranks 1–4) are red, and stroma aspect hexagons (ranks -1 to -4) are green. Color intensity reflects distance from the tumor edge, with rank colors matching those in panel D. (D) Box-and-whisker plot showing CD8+ cell density (cells/mm²) across the IZ, with ranks from -4 to 4 on the x-axis. (E, F, G, H) Ki67 percentage, Ki67 entropy, CD8 mean in the tumor aspect of the IZ (CD8_m_T), and CD8 immunodrop (CD8_ID) values are marked by white circle-cross symbols. The red vertical line marks prognostic cutoff values. Gray shading of bars indicates prognosis, with brighter shades indicating better prognosis and darker shades indicating worse. BCSS: breast cancer-specific survival.

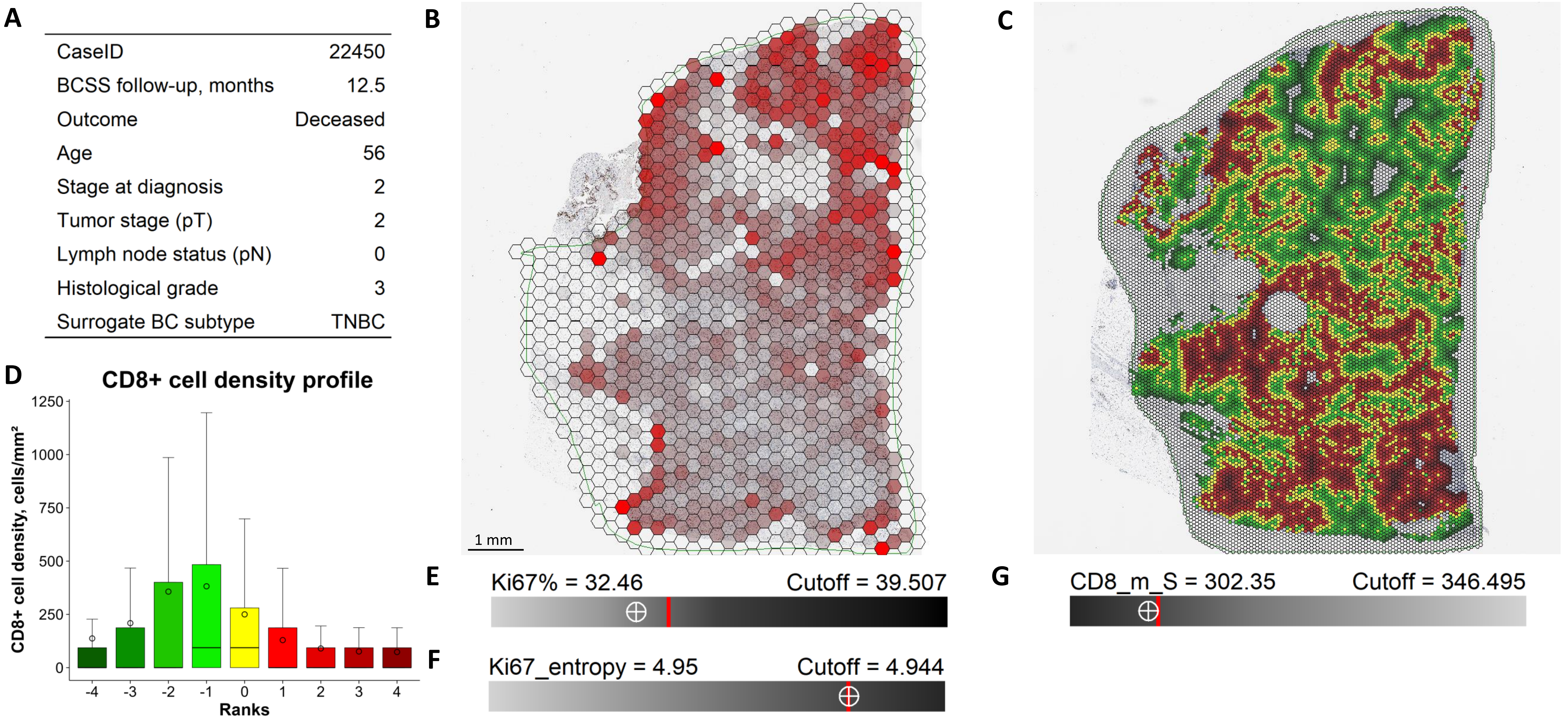

S6 Fig. Extracted Ki67-intratumoral heterogeneity and CD8-immunogradient indicators in breast cancer (BC) case. (A) Clinicopathological indicators for a BC patient. (B) Whole-slide image of BC tissue stained for Ki67, with a hexagonal grid overlaid. Ki67-positive cell percentages within each hexagon are color-coded in red shades to show regions of higher positivity and spatial heterogeneity. (C) Whole-slide image of BC tissue stained for CD8+ cells, with a hexagonal grid and an interface zone (IZ) of 9 hexagons in width extracted. Tumor edge hexagons are yellow (rank 0), tumor aspect hexagons (ranks 1–4) are red, and stroma aspect hexagons (ranks -1 to -4) are green. Color intensity reflects distance from the tumor edge, with rank colors matching those in panel D. (D) Box-and-whisker plot showing CD8+ cell density (cells/mm²) across the IZ, with ranks from -4 to 4 on the x-axis. (E, F, G) Ki67 percentage, Ki67 entropy, and CD8 mean in the stroma aspect of the IZ (CD8_m_S) values are marked by white circle-cross symbols. The red vertical line marks prognostic cutoff values. Gray shading of bars indicates prognosis, with brighter shades indicating better prognosis and darker shades indicating worse. BCSS: breast cancer-specific survival, TNBC: triple-negative breast cancer.

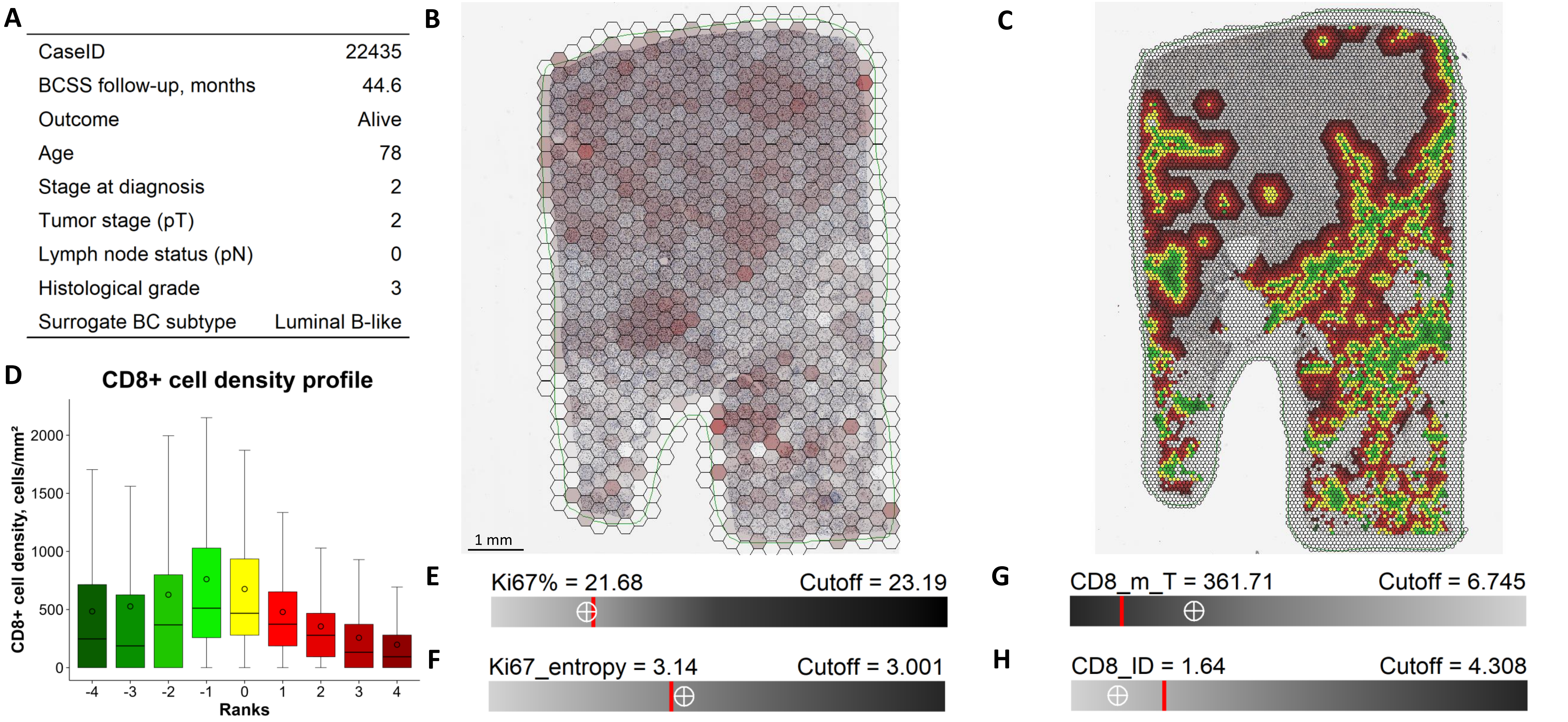

S7 Fig. Extracted Ki67-intratumoral heterogeneity and CD8-immunogradient indicators in breast cancer (BC) case. (A) Clinicopathological indicators for a BC patient. (B) Whole-slide image of BC tissue stained for Ki67, with a hexagonal grid overlaid. Ki67-positive cell percentages within each hexagon are color-coded in red shades to show regions of higher positivity and spatial heterogeneity. (C) Whole-slide image of BC tissue stained for CD8+ cells, with a hexagonal grid and an interface zone (IZ) of 9 hexagons in width extracted. Tumor edge hexagons are yellow (rank 0), tumor aspect hexagons (ranks 1–4) are red, and stroma aspect hexagons (ranks -1 to -4) are green. Color intensity reflects distance from the tumor edge, with rank colors matching those in panel D. (D) Box-and-whisker plot showing CD8+ cell density (cells/mm²) across the IZ, with ranks from -4 to 4 on the x-axis. (E, F, G, H) Ki67 percentage, Ki67 entropy, CD8 mean in the tumor aspect of the IZ (CD8_m_T), and CD8 immunodrop (CD8_ID) values are marked by white circle-cross symbols. The red vertical line marks prognostic cutoff values. Gray shading of bars indicates prognosis, with brighter shades indicating better prognosis and darker shades indicating worse. BCSS: breast cancer-specific survival.

A

CaseID	22217
BCSS follow-up, months	109.9
Outcome	Alive
Age	81
Stage at diagnosis	1
Tumor stage (pT)	1
Lymph node status (pN)	0
Histological grade	3
Surrogate BC subtype	TNBC

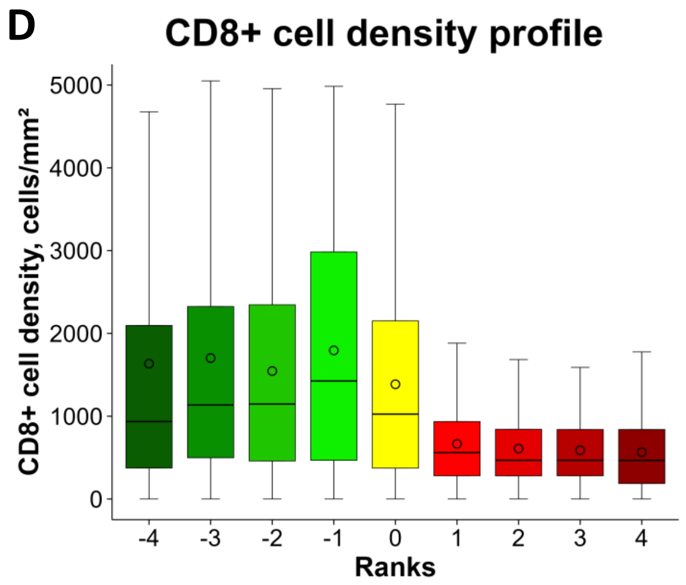

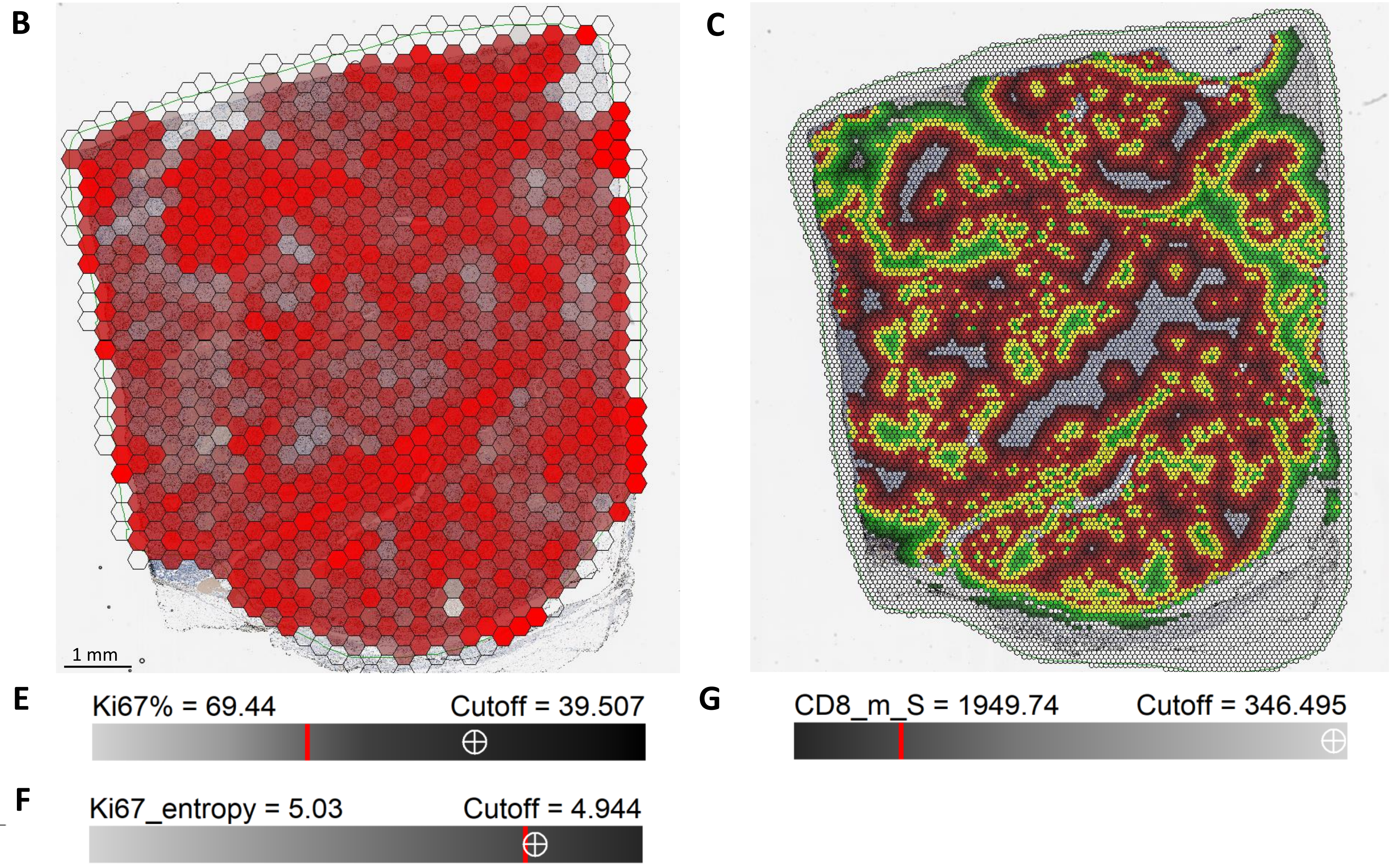

S8 Fig. Extracted Ki67-intratumoral heterogeneity and CD8-immunogradient indicators in breast cancer (BC) case. (A) Clinicopathological indicators for a BC patient. (B) Whole-slide image of BC tissue stained for Ki67, with a hexagonal grid overlaid. Ki67-positive cell percentages within each hexagon are color-coded in red shades to show regions of higher positivity and spatial heterogeneity. (C) Whole-slide image of BC tissue stained for CD8+ cells, with a hexagonal grid and an interface zone (IZ) of 9 hexagons in width extracted. Tumor edge hexagons are yellow (rank 0), tumor aspect hexagons (ranks 1–4) are red, and stroma aspect hexagons (ranks -1 to -4) are green. Color intensity reflects distance from the tumor edge, with rank colors matching those in panel D. (D) Box-and-whisker plot showing CD8+ cell density (cells/mm²) across the IZ, with ranks from -4 to 4 on the x-axis. (E, F, G) Ki67 percentage, Ki67 entropy, and CD8 mean in the stroma aspect of the IZ (CD8_m_S) values are marked by white circle-cross symbols. The red vertical line marks prognostic cutoff values. Gray shading of bars indicates prognosis, with brighter shades indicating better prognosis and darker shades indicating worse. BCSS: breast cancer-specific survival, TNBC: triple-negative breast cancer.

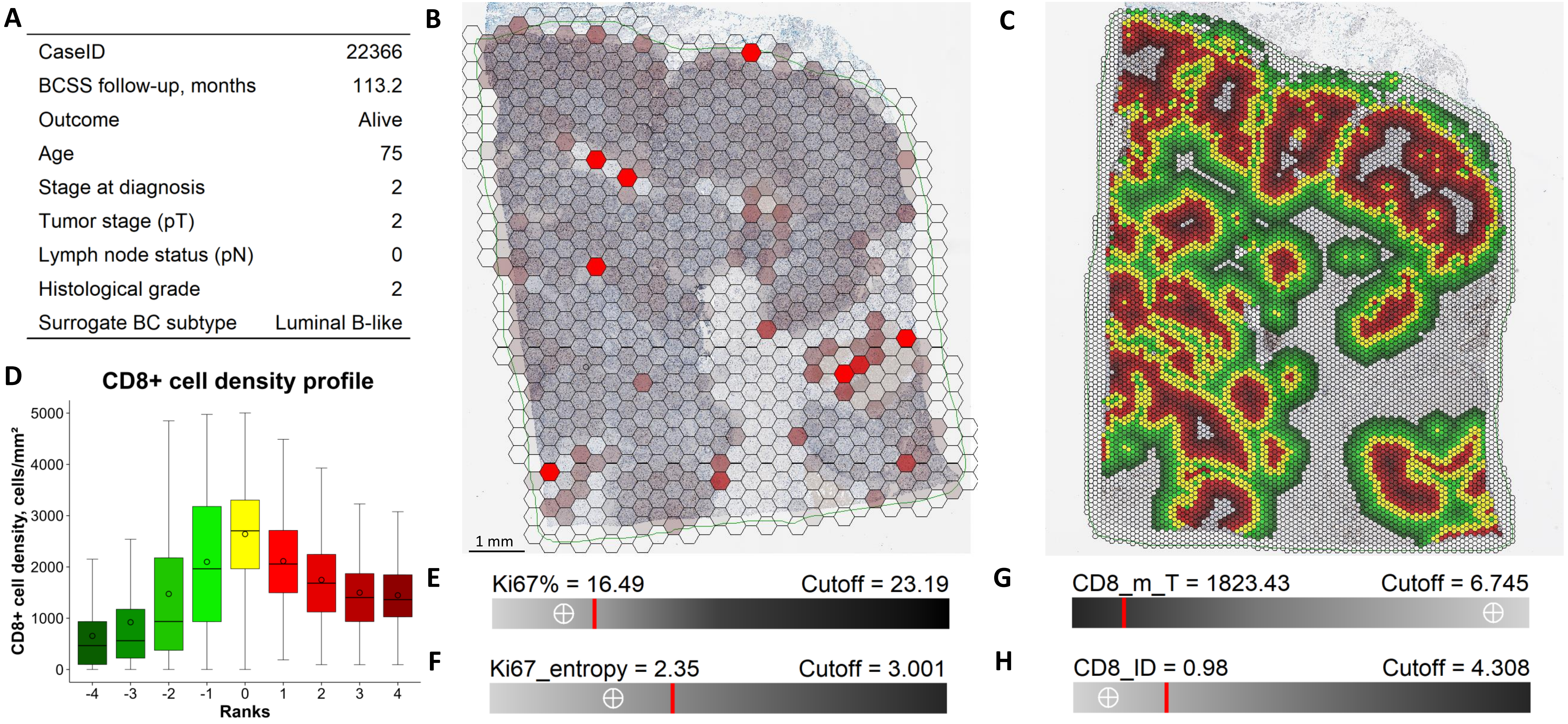

S9 Fig. Extracted Ki67-intratumoral heterogeneity and CD8-immunogradient indicators in breast cancer (BC) case. (A) Clinicopathological indicators for a BC patient. (B) Whole-slide image of BC tissue stained for Ki67, with a hexagonal grid overlaid. Ki67-positive cell percentages within each hexagon are color-coded in red shades to show regions of higher positivity and spatial heterogeneity. (C) Whole-slide image of BC tissue stained for CD8+ cells, with a hexagonal grid and an interface zone (IZ) of 9 hexagons in width extracted. Tumor edge hexagons are yellow (rank 0), tumor aspect hexagons (ranks 1–4) are red, and stroma aspect hexagons (ranks -1 to -4) are green. Color intensity reflects distance from the tumor edge, with rank colors matching those in panel D. (D) Box-and-whisker plot showing CD8+ cell density (cells/mm²) across the IZ, with ranks from -4 to 4 on the x-axis. (E, F, G, H) Ki67 percentage, Ki67 entropy, CD8 mean in the tumor aspect of the IZ (CD8_m_T), and CD8 immunodrop (CD8_ID) values are marked by white circle-cross symbols. The red vertical line marks prognostic cutoff values. Gray shading of bars indicates prognosis, with brighter shades indicating better prognosis and darker shades indicating worse. BCSS: breast cancer-specific survival.

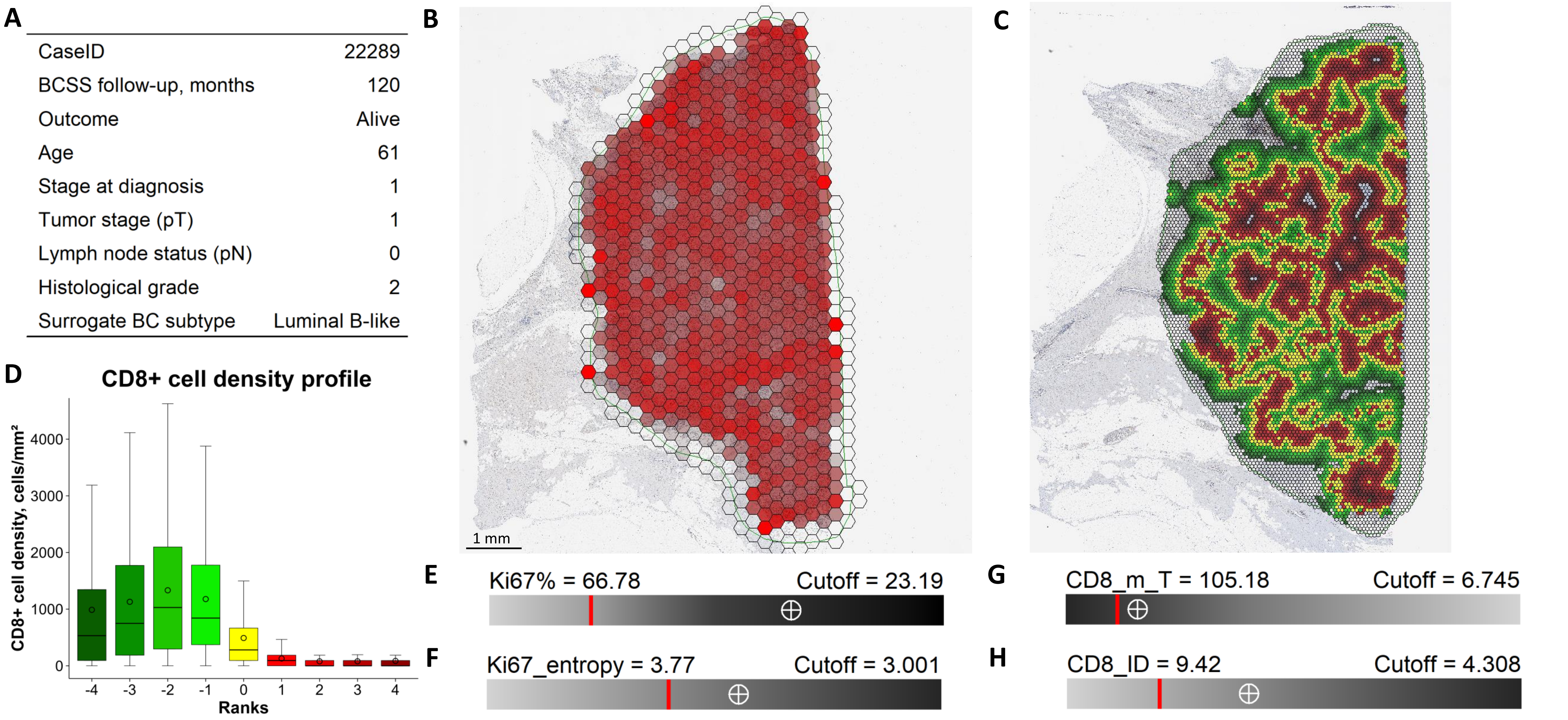

S10 Fig. Extracted Ki67-intratumoral heterogeneity and CD8-immunogradient indicators in breast cancer (BC) case. (A) Clinicopathological indicators for a BC patient. (B) Whole-slide image of BC tissue stained for Ki67, with a hexagonal grid overlaid. Ki67-positive cell percentages within each hexagon are color-coded in red shades to show regions of higher positivity and spatial heterogeneity. (C) Whole-slide image of BC tissue stained for CD8+ cells, with a hexagonal grid and an interface zone (IZ) of 9 hexagons in width extracted. Tumor edge hexagons are yellow (rank 0), tumor aspect hexagons (ranks 1–4) are red, and stroma aspect hexagons (ranks -1 to -4) are green. Color intensity reflects distance from the tumor edge, with rank colors matching those in panel D. (D) Box-and-whisker plot showing CD8+ cell density (cells/mm²) across the IZ, with ranks from -4 to 4 on the x-axis. (E, F, G, H) Ki67 percentage, Ki67 entropy, CD8 mean in the tumor aspect of the IZ (CD8_m_T), and CD8 immunodrop (CD8_ID) values are marked by white circle-cross symbols. The red vertical line marks prognostic cutoff values. Gray shading of bars indicates prognosis, with brighter shades indicating better prognosis and darker shades indicating worse. BCSS: breast cancer-specific survival.

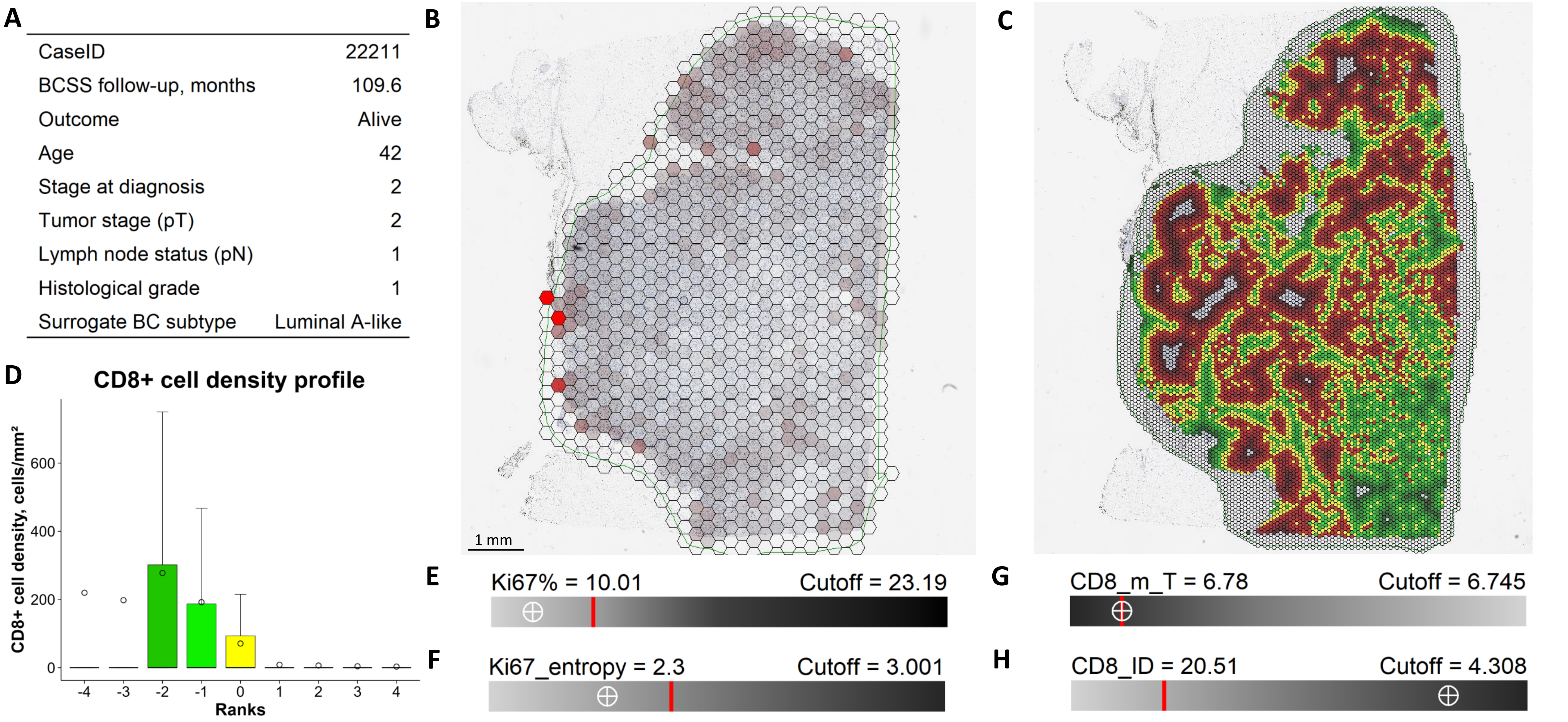

S11 Fig. Extracted Ki67-intratumoral heterogeneity and CD8-immunogradient indicators in breast cancer (BC) case. (A) Clinicopathological indicators for a BC patient. (B) Whole-slide image of BC tissue stained for Ki67, with a hexagonal grid overlaid. Ki67-positive cell percentages within each hexagon are color-coded in red shades to show regions of higher positivity and spatial heterogeneity. (C) Whole-slide image of BC tissue stained for CD8+ cells, with a hexagonal grid and an interface zone (IZ) of 9 hexagons in width extracted. Tumor edge hexagons are yellow (rank 0), tumor aspect hexagons (ranks 1–4) are red, and stroma aspect hexagons (ranks -1 to -4) are green. Color intensity reflects distance from the tumor edge, with rank colors matching those in panel D. (D) Box-and-whisker plot showing CD8+ cell density (cells/mm²) across the IZ, with ranks from -4 to 4 on the x-axis. (E, F, G, H) Ki67 percentage, Ki67 entropy, CD8 mean in the tumor aspect of the IZ (CD8_m_T), and CD8 immunodrop (CD8_ID) values are marked by white circle-cross symbols. The red vertical line marks prognostic cutoff values. Gray shading of bars indicates prognosis, with brighter shades indicating better prognosis and darker shades indicating worse. BCSS: breast cancer-specific survival.
